# Biomarkers in Lupus Nephritis: An Evidence-Based Comprehensive Review

**DOI:** 10.3390/life15101497

**Published:** 2025-09-23

**Authors:** Alexandra Vrabie, Bogdan Obrișcă, Bogdan Marian Sorohan, Gener Ismail

**Affiliations:** 1 Department of Nephrology, “Carol Davila” University of Medicine and Pharmacy, 050474 Bucharest, Romania; obriscabogdan@yahoo.com (B.O.); bogdan.sorohan@yahoo.com (B.M.S.); gener732000@yahoo.com (G.I.); 2 Department of Nephrology, Fundeni Clinical Institute, 022328 Bucharest, Romania; 3 Department of Kidney Transplantation, Fundeni Clinical Institute, 022328 Bucharest, Romania

**Keywords:** systemic lupus erythematosus, lupus nephritis, biomarkers, diagnosis, prognosis, renal flare, monitoring

## Abstract

**Background and Objectives**: Lupus nephritis (LN) is a major cause of mortality and morbidity in patients with systemic lupus erythematosus (SLE). Biomarkers derived from blood, urine, and multi-omics techniques are essential for enabling access to less invasive methods for LN evaluation and personalized precision medicine. **Materials and Methods**: The purpose of this work was to review the studies that addressed the potential role of urinary and serological biomarkers for the diagnosis, disease activity, response to treatment, and renal outcome of adult patients with LN, published over the past decade, and summarize their results with a particular emphasis being directed towards the available traditional tools. **Results**: Traditional biomarkers used for the diagnosis and surveillance of LN are proteinuria, urinary sediment, estimated glomerular filtration rate (eGFR), anti-double-stranded deoxyribonucleic acid (anti-dsDNA), anti-C1q, and serum complement levels. Anti-dsDNA, serum C3, and proteinuria are the conventional biomarkers with the strongest clinical evidence, with overall moderate ability in predicting LN from non-renal SLE, disease activity, renal flares, response to therapy, and prognosis. The last decade has brought significant progress in our understanding regarding the pathogenesis of LN and, consequently, several molecules, either alone or in combination panels, have emerged as potential novel biomarkers, some of them outperforming conventional biomarkers. Promising results have been suggested for urinary activated leukocyte cell adhesion molecule (ALCAM), soluble cluster of differentiation 163 (CD163), C-X-C motif chemokine ligand 10 (CXCL10), monocyte chemoattractant protein 1 (MCP-1), neutrophil gelatinase-associated lipocalin (NGAL), tumor necrosis factor-like weak inducer of apoptosis (TWEAK), and vascular cell adhesion molecule 1 (VCAM-1). **Conclusions**: Despite the intensive research of the last decade, no novel biomarker has entered clinical practice, and we continue to rely on traditional biomarkers to assess non-invasively LN and guide its treatment. Novel biomarkers should be validated in multiple longitudinal independent cohorts, compared with conventional biomarkers, and integrated with renal histology information in order to optimize the management of LN patients.

## 1. Introduction

Lupus nephritis (LN) occurs in up to 60% of patients with systemic lupus erythematosus (SLE) and is the most important predictor of morbidity and mortality among these patients [1,2]. Despite the important progress seen in recent years with the increased knowledge of the genetic basis and the pathophysiology of LN and the advent of newer treatment strategies, a significant proportion of patients still fail to attain a complete renal response (CRR) after 2 years of therapy, and up to 35% of the patients may subsequently develop flares of activity [2,3,4]. Moreover, 30% of the patients still progress to end-stage kidney disease (ESKD) [5].

A kidney biopsy is fundamental for the diagnosis and management of LN [3]. However, it is an invasive maneuver that describes an evolving pathogenic process based on only one time point, which led the nephrology community in the direction of searching for clinical scenarios in which a repeat kidney biopsy approach is needed, but also for non-invasive biomarkers that can be easily assessed longitudinally [4,6,7]. Traditional biomarkers used to assess LN activity and prognosis are proteinuria, urinary sediment, estimated glomerular filtration rate (eGFR), anti-double-stranded deoxyribonucleic acid (anti-dsDNA), anti-C1q, and serum complement levels [4,8,9,10]. Unfortunately, these current laboratory biomarkers have limited ability to differentiate renal activity from chronic lesions without histological examination and to predict early-oncoming flares of the disease [8,9].

A non-invasive biomarker-based renal assessment that reflects renal pathology may improve routine clinical diagnosis and monitoring of flares and their management by matching in real time the treatment decisions with the kidney status in a longitudinal approach, equilibrating the fragile balance between over- and under-treatment that a clinician has to always keep in mind [6,11,12]. Recently, the intensive research oriented towards precision medicine, with the increasing use of multi-omic techniques, has provided a growing body of evidence for novel non-invasive biomarkers [9,13]. Although several molecules, either alone or in combination panels, have emerged as potential novel biomarkers in LN, no novel biomarker has entered clinical practice in LN. Their performance remains to be validated in multiple longitudinal independent cohorts and compared with traditional biomarkers to enter clinical practice [14].

The purpose of this work is to provide an evidence-based update of the last decade’s literature regarding non-invasive biomarkers in LN, with a particular emphasis being directed towards the available traditional tools.

## 2. Materials and Methods

A literature search of PubMed and Embase electronic databases was performed from 1/2014 to 9/2025 to identify all studies that addressed the potential role of urinary and/or serological biomarkers for the diagnosis, disease activity, prognosis, and response to treatment of adult SLE patients with LN. A combination of the following words was used: “lupus nephritis” and “biomarkers”. The inclusion criterion of the studies was the urinary and/or serological testing of traditional and novel biomarkers in SLE patients with LN. The exclusion criteria of the studies were the following: studies with non-laboratory biomarkers, with inadequate cohorts (with other or mix of diagnoses), with not enough information about the patients, with no abstract availability, in languages other than English, on pediatric LN, case series, case reports, animal studies, preclinical studies, reviews, congress abstracts, and retracted publications. Disease activity in SLE patients was quantified in the identified studies based on the SLE Disease Activity Index (SLEDAI) score, European Consensus Lupus Activity Measurement (ECLAM) index, or British Isles Lupus Assessment Group (BILAG) index (the information is found in the tables in the “Disease Activity Evaluation” section for each study). In the analyzed studies, the SLEDAI score was calculated based either on the original SLEDAI or on the modified version named SLEDAI 2000 (SLEDAI-2K), as written in the methods of the studies. All identified articles were analyzed by two reviewers (A.V. and B.O.) for inclusion and exclusion criteria, and a third reviewer (G.I.) coordinated the review process. Discrepencies between the two primary reviewers were resolved by consensus; if consensus could not be achieved, the disagreements were discussed with the third reviewer to arrive at a consensus.

## 3. Lupus Nephritis Biomarkers

Kidney Disease Improving Global Outcomes (KDIGO) 2024 Clinical Practice Guideline for the Management of LN recommends that patients with SLE be tested at the disease presentation, as regular monitoring and in case of a suspicion of a SLE flare, for the following well-established, traditional kidney disease-related and immunoserologic markers: serum creatinine, urinalysis with dipstick and sediment, spot urine protein-creatinine ratio (UPCR), anti-dsDNA, and complement fractions [15].

SLEDAI-2K includes the following kidney disease-related markers: the presence of heme-granular or red blood cell (RBC) urinary casts, hematuria defined as >5 RBC/high power field, proteinuria defined as >0.5 g/day and pyuria defined as >5 white blood cells (WBC)/high power field, and the following immunoserologic markers: low CH50, C3 or C4 and increased deoxyribonucleic acid (DNA) binding [16]. BILAG-2024 Index includes the following renal manifestations occurring in the last 4 weeks: values of systolic and diastolic blood pressure, presence of accelerated hypertension, values of dipstick protein, urine albumin-creatinine ratio (ACR), UPCR, and 24 h urine protein, presence of nephrotic syndrome, value of serum creatinine and calculated GFR, presence of active urinary sediment and active nephritis [17].

The 2019 Update of the Joint European League Against Rheumatism (EULAR) and European Renal Association- European Dialysis and Transplant Association recommendations for the management of LN include testing for anti-C1q autoantibodies in patients with suspected LN whenever available [10]. The contribution of anti-C1q autoantibodies to LN pathogenesis was suggested by previous studies that have shown that the infusion of anti-C1q antibodies into the kidney of mice that have low levels of immune complex deposition can lead to renal inflammation secondary to the activation of the classical complement pathway [18,19]. Also, a target for anti-C1q antibodies is the C1q complexed with the damaged cells within the kidney [20]; additionally, these antibodies may interfere with the clearance of immune complexes and apoptotic material through C1q, similarly to C1q deficiency [18,21].

Traditionally, clinical response is defined in clinical trials and practice based on proteinuria reduction and kidney function improvement or stabilization [14,15]. Table 1 summarizes the definitions of CRR used in LN landmark randomized controlled trials.

The definition of complete response includes a reduction in UPCR < 0.5 g/g from a 24 h urine collection [15]. The post hoc analysis of the Euro-Lupus Nephritis and MAINTAIN Nephritis trials supports the use of the primary efficacy renal response (PERR), defined as a PCR ≤ 0.7 g/g [15,40,41]. Regarding kidney function evaluation, the criterion used in the CRR definition is a kidney function ±10%–15% of its baseline value, whereas in the PERR, it is required that the eGFR be no less than 20% below the baseline value or to be ≥60 mL/min/1.73m^2^ [15]. In clinical trials, the response to therapy is evaluated at 6–12 months, but in clinical practice, the rate of improvement of these clinical parameters varies among patients, and accordingly, the KDIGO Work Group suggests a less tight timeframe for assessing the treatment response, allowing 18–24 months in patients who are constantly showing signs of improvement [15].

The 2024 American College of Rheumatology (ACR) Guideline of the Screening, Treatment, and Management of LN recommends that the surveillance of LN activity should include testing of proteinuria at least every 3 months in patients who have not achieved CRR and every 3–6 months in those who are in sustained clinical remission. Also, it is advised to measure serum complement and anti-dsDNA antibody periodically at every visit in the clinic, but no more than once per month, and changes in their levels should raise attention with further close monitoring, without the need for preemptive treatment in the absence of clinical signs of flares [42].

**Figure 1:**
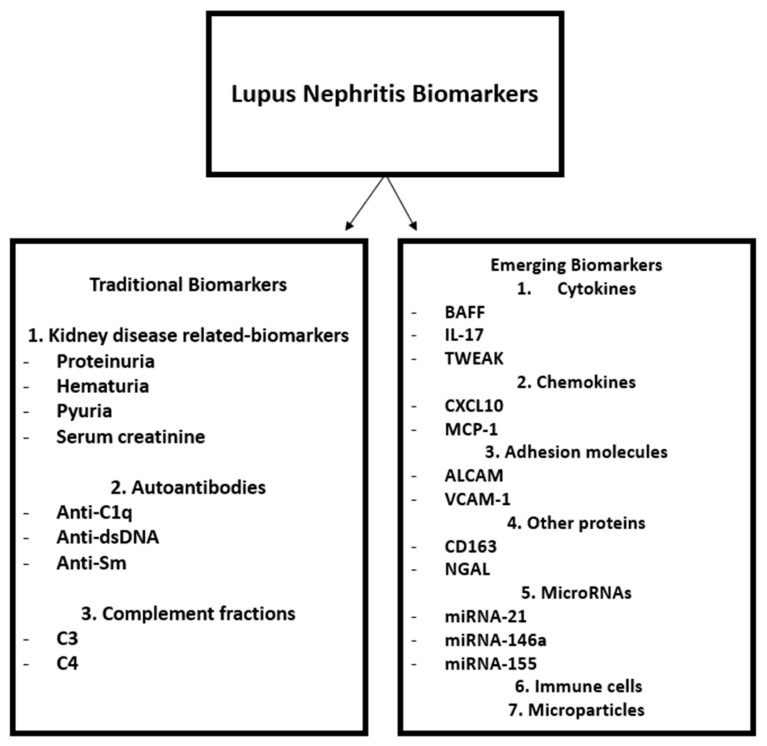
Lupus Nephritis Biomarkers.

We synthesized in Figure 1 the conventional and emerging biomarkers in LN, which will be further discussed throughout the article.

### 3.1. Kidney Disease Related-Biomarkers

#### 3.1.1. Urine Parameters

Proteinuria is a key feature of LN and the most used marker in screening and monitoring LN. The KDIGO 2024 Clinical Practice Guideline for the Management of LN recommends initial assessment of proteinuria by dipstick or spot UPCR with further confirmation by a 24 h urine collection in case of dipstick-protein ≥ 2+ (any specific gravity), 1+ (low specific gravity), or spot UPCR > 500 mg/g [9,15,18].

Results from different research groups support the diagnostic role of proteinuria for distinguishing LN from non-renal SLE patients (area under the curve (AUC) 0.92–0.93) [43,44]. Despite the evidence of proteinuria being a good predictor of LN disease activity based on SLEDAI score (AUC 0.89–0.99) or by the presence of proliferative LN (AUC 0.748) [45,46,47,48], a growing number of repeat biopsy studies have shown a discordance between clinical and expected histological findings [14,49]. Malvar et al. evaluated 69 patients with proliferative LN by per-protocol biopsy at 6 months after induction therapy and found that one-third of patients who had achieved CRR still had persistently high histologic activity defined as a National Institutes of Health (NIH) activity index (AI) of 5 or higher at 6 months [50]. Also, in the same cohort, from the 13 patients who reached complete histologic remission (defined as AI of 0) at 6 months, still had proteinuria of more than 500 mg/day [50]. Parodis et al. investigated a cohort of 42 patients by per-protocol biopsy after a median of 24.3 months and found that 23.8% of them were still active histologically (AI > 3) despite a urinary PCR of less than 1 g/g, whereas 7.1% of them had persistently UPCR ≥ 1 g/g despite having an AI score ≤ 3 [51]. A limitation regarding the current parameters used for the evaluation of response to treatment, represented by proteinuria and renal function, is that they can not differentiate between persistent intrarenal activity, chronic lesions, and other non-immunologic factors contributing to nephron loss [14,15,52].

Prevention and early identification of LN flares are key treatment targets, as the relapse of LN is a strong predictor of poor long-term kidney outcome [52,53,54]. Proteinuria > 0.8 g/24 h at 12 months was associated with a higher risk of flare (odds ratio (OR) 4.12) and a shorter time to renal flare (OR 2.57) in a cohort of 100 proliferative LN [55]. Fatemi et al. found that proteinuria > 0.5 g/24 h at first visit is a good predictor of LN flare, showing a negative predictive value (NPV) of 85% in their analysis during a follow-up of 18 months [47].

Also, recent studies showed that a low baseline level of proteinuria (0.1–0.87 g/24 h) is predictive of CRR after 6 months of treatment with an OR of 4.3 [56] and a UPCR < 1.5 g/g at month 6 can predict CRR by month 12 with a sensitivity (Sn) of 86% and a specificity (Sp) of 81% [6].

Moreover, proteinuria proved to be a good predictor of renal outcome in several studies. In the Euro-Lupus Nephritis Trial, proteinuria of <0.8 g/24 h at 12 months had an Sn of 81% and a Sp of 78% for predicting good long-term renal function [40]. The long-term data from the MAINTAIN Nephritis Trial validate the early target of proteinuria of less than <0.7 g/24 h for a good long-term renal outcome, achieving a Sn of 71% and a Sp of 75%, with a high positive predictive value (PPV) of 94%, but a low NPV of 31%. The low NPV suggests that two-thirds of these patients will still experience a long-term good renal outcome despite not achieving the early target after 12 months of treatment [41]. Another study showed that proteinuria > 0.8 g/day at 12 months can predict the development of stage 3–4 chronic kidney disease (CKD) or end-stage kidney disease (ESKD) after a median follow-up of 100 months with an OR 10.8 [55]. In the study conducted by Petri, proteinuria at diagnosis was predictive of renal failure, defined as the need for dialysis or kidney transplant within 20 years, with an adjusted relative risk (RR) of 2.75 [57].

The presence of an active urinary sediment correlates with LN activity in several studies [45,46,58]; Jakiela et al. found an AUC of 0.92 for RBC (≥10 cells/high power field), 0.91 for granular casts, and 0.75 for WBC for predicting LN disease activity based on the SLEDAI score [45]. As a prognosis marker, the absence of hematuria (red blood cells ≤ 5/high power field) at 12 months after randomization in the Euro-Lupus Nephritis Trial showed low Sn and Sp (62%, 64%, respectively) for predicting good long term renal function and its addition to proteinuria alone or proteinuria and serum creatinine lowered their Sn, leading to misclassification of more than 50% of the patients as trial non-responders even though they had a good renal outcome [40].

#### 3.1.2. Serum Parameters

Results from different research groups support the role of increased serum creatinine (AUC 0.76–0.82) for the diagnosis of patients with LN when compared with those with non-renal SLE [59,60]. Different research groups found a modest role of serum creatinine as a disease activity biomarker, being able to differentiate active from inactive LN based on SLE disease activity index (SLEDAI) score with an AUC of 0.62–0.68, Sn of 84.6% and low Sp of 35% [46,61].

Higher values of serum creatinine at baseline can predict the renal outcome (hazard ratio (HR) 2.1 for ESKD, HR 1.17 for sustained 30% decline of eGFR, adjusted HR 4.655 for death and doubling of serum creatinine/ESKD) [62,63,64]. In the Euro-Lupus Nephritis Trial, the cut-off of serum creatinine of ≤0.8 mg/dL at 12 months was as specific as proteinuria < 0.8 g/day at 12 months (81–83%), but showed a lower Sn (58%) in predicting good long-term renal outcome defined as a serum creatinine ≤ 1 mg/dL after 7 years of follow-up [40]. Raising the cut-off of serum creatinine to ≤1 mg/dL raised the Sn at 90% at the expense of a low Sp (48%) for predicting renal outcome [40].

Table 2 summarizes the performances of kidney disease-related markers investigated in the studies in the last ten years.

### 3.2. Autoantibodies

Traditional serologic parameters are represented by anti-dsDNA and anti-C1q, and guidelines suggest using them in patients with SLE and suspected LN, and for LN monitoring [10].

Results from different studies support the overall satisfactory ability of anti-dsDNA to predict LN in SLE patients with an AUC of 0.6–0.89 and NPV of 81–100% [43,61,76,77,78,79,80,81,82,83,84,85,86,87,88,89]. Also, anti-dsDNA demonstrated moderate overall ability in predicting the presence of proliferative LN with an AUC of 0.70–0.83 and in distinguishing active from inactive LN based on SLEDAI or BILAG scores (AUC 0.66–0.88, OR 3.86–4.8) [45,59,61,83,88,89,90,91,92,93,94,95].

Fasano et al. found that a cut-off of more than 10 IU for anti-dsDNA could predict a subsequent renal flare with an AUC of 0.85 and HR of 21.67 [96]. Furthermore, Himbert et al. highlight the potential role of the baseline immunoglobulin E (IgE) isotype of anti-dsDNA antibodies for predicting LN flares during the 24 months of follow-up in the WIN-IgE study (Sn 40%, Sp 90%) [97]. Another novel finding is represented by the anti-dsDNA autoantibodies secreting cells enzyme-linked immunosorbent spot (SLE-ELISpot) detected in blood samples, a high result being associated with an increased HR for renal flare during the 12-month follow-up (HR 6.5) [98].

Vriese et al. proposed including as an aim of the LN treatment also the immunological remission in LN (defined as negative anti-dsDNA) to decrease the low-grade generation and deposition of immune complexes during clinical remission and withdrawing the therapy only after at least 1 year of sustained immunological remission [14]. The inclusion of this stringent goal, as in the previously proposed definitions of remission in SLE (DORIS), complete remission off-treatment criteria [99]. It remains an open question, taking into account the need for lifelong immunosuppression and the cumulative drug-induced damage in a subset of LN patients.

In the study conducted by Kim, the baseline titer of anti-dsDNA predicted no renal response to treatment at 6 months with an AUC of 0.73. In the same study, C3 hypocomplementemia performed better in predicting the absence of renal response at 6 months, demonstrating an AUC of 0.84 compared with the metrics of the anti-dsDNA and C4 fraction, with renal response being defined as complete if the UPCR was <500 mg/g and the eGFR was normal or within 10% of normal eGFR, and partial if proteinuria declined by ≥50% and the eGFR was normal or within 10% of normal eGFR [100]. Mejia-Vilet et al. evaluated the ability of the 6-month titers of anti-dsDNA and C3 in predicting the CRR at 12 months. In their cohort, the disappearance of the anti-dsDNA antibodies and C3 normalization at 6 months can predict the response to therapy at 12 months with a Sn of 70% and Sp of 56% and a Sn of 70% and Sp of 67%, respectively [6].

Whittal-Garcia et al. found in a cohort of 92 active LN patients a HR for sustained 50% decline in eGFR and for ESKD of 1.97 and 1.89, respectively, for every increase in 50 IU/L of anti-dsDNA at baseline [63].

Also, despite the lack of standardized laboratory assays [14], results from multiple cohorts suggest the potential role of anti-C1q for identifying LN from non-renal SLE with an AUC of 0.64–0.84 and variable Sn (47–100%) and Sp (47.7–92%) [62,81,82,83,89,92,101,102,103,104,105]. Anti-C1q as a disease activity biomarker has been evaluated in different studies, showing moderate ability in predicting the presence of histological disease activity with an AUC of 0.71–0.73 [89,90] and clinical disease activity based on SLEDAI or BILAG scores, with an AUC of 0.72–0.76 [83,92,106].

Fatemi et al. found in a prospective cohort of SLE patients that anti-C1q positivity could predict renal flares with a low PPV of 35%, but a high NPV of 93% and adding low C3 could add more accuracy by raising the PPV to 60% [47]. In a study conducted by Birmingham, there was a rise in the titer of anti-C1q antibodies at the time of LN flare only in those patients who had also anti-C3b positivity (*p* = 0.02) [102].

Moreover, the analysis conducted by Pang showed that the positivity of anti-C1q (A08 epitope) is a risk factor for death and doubling of serum creatinine or ESKD after a median follow-up of 42 months, with an HR of 3.9 and an adjusted HR of 1.2 [62].

Table 3 summarizes the performances of serum antibodies as biomarkers investigated in the studies in the last ten years.

### 3.3. Complement

A core feature of the pathophysiology of the glomerular injury in SLE is the in vivo activation of the complement system by the immune complexes, which further initiates the intrarenal inflammation and injury [18,53]. The following studies showed the potential diagnostic role for C3 hypocomplementemia (AUC 0.65–0.91, NPV 71–95%, OR 4–6.4) [43,61,76,78,84,87,88,89,111,112]. In the study conducted by Ishizaki, low C3 (<65 mg/dL) proved an OR of 39 with an Sn of 78% and an Sp of 92% for the diagnosis of “silent” LN in patients with SLE without abnormal urinalysis or renal impairment at the time of kidney biopsy [107]. Low C4 as a diagnostic marker of LN was evaluated in a series of studies and proved to have a modest role (AUC 0.619–0.71, NPV 70%) [43,76,86,87,112]. The activation products of complement are currently not measured in standard clinical practice because these assays need special conditions of handling to obtain a valid value [18]. Martin et al. found that high C4d can be a potential marker for discriminating LN from non-renal SLE with an OR of 0.8 and an AUC of 0.71; moreover, the ratio of C4d to C4 seems to be a better predictor for LN with an OR of 13.1, AUC 0.76, and a Sn of 83% with a Sp of 73% [111].

Low levels of C3 are a good predictor of active LN in several studies (AUC 0.82–0.89, Sn 74–100%, Sp 64.7–84.21%) [45,61,88,113,114]. As a marker of histological activity, low C3 could differentiate between proliferative and non-proliferative LN or class V LN with an AUC of 0.70–0.77 [94,95,115] and could weakly predict an AI more than 8 with an AUC of 0.68 [116]. Also, C4 hypocomplementemia was evaluated in SLE cohorts regarding its potential disease activity predictor with the following metrics (AUC 0.68–0.88, Sn 81.3%, Sp 88.2%) [45,48,113]. Moreover, C4d correlated significantly with histological activity (correlation with AI-r 0.37, *p* = 0.002) [111].

Hypocomplementemia may predict renal flares with an AUC of 0.76 for low C3 and 0.82 for low C4 and an NPV of 100% for both [96]. Also, Ruchakorn et al. found an OR of 2.5 for low C3 [117], and Buyon et al. found an adjusted OR of 5.6 for low C4 in predicting renal flares [118].

Also, low levels of C3 seem to be a potential prognostic biomarker in several studies. Rossi et al. found that persistent isolated C3 hypocomplementemia 6 months after kidney biopsy is a risk factor for death with a HR of 2.56 and for ESKD with an adjusted HR of 3.41 [119]. Moreover, Petri et al. found that a low C3 ever is predictive of renal failure in the next 20 years with an adjusted RR of 2.0 in the Hopkins Lupus Cohort [57].

Table 4 summarizes the performances of complement tests investigated in the studies in the last ten years.

### 3.4. Emerging Biomarkers

#### 3.4.1. Cytokines

The clinical heterogeneity of SLE is accompanied by complex cytokine pathways that may dominate in different patients, organ systems affected, and stages of the disease [18]. Cytokines are small soluble mediators that are induced by triggers of both innate and adaptive immune response, inadequate production leading to autoimmunity, inflammation, and ultimately tissue injury in LN patients [18]. The cytokines identified in recent studies as promising biomarkers in LN are represented by B lymphocyte stimulator (BLyS), interleukin (IL)-17, and tumor necrosis factor-like weak inducer of apoptosis (TWEAK).

BlyS, also known as B-cell activating factor (BAFF), has an important role in B-cell maturation and survival [18,120]. Belimumab is a human monoclonal antibody that inhibits BAFF and now is included in the recommended approach for patients with proliferative LN by KDIGO 2024 Clinical Practice Guideline for the Management of LN, both as induction therapy in combination with glucocorticoids and standard dose mycophenolic acid analogs or reduced-dose cyclophosphamide and as maintenance therapy in combination with low-dose glucocorticoids and mycophenolic acid analogs or azathioprine, based on efficacy demonstrated in BLISS-LN and the open label extension trials [15,35,121]. Three recent studies found that urinary BAFF can discriminate LN from non-renal SLE with an AUC of 0.79–0.93 [112,122], with a low Sn of 20% and a high Sp of 91% [123], outperforming the conventional markers analyzed concomitantly (AUC 0.45–0.66 for anti-dsDNA, 0.57–0.81 for serum C3, and 0.61–0.63 for serum C4) [112,122]. Moreover, Parodis et al. found that the baseline levels of <1.5 ng/mL of serum BAFF can predict clinical response with a PPV of 92% and histological response with a PPV of 84% in a cohort of 52 proliferative LN patients [120].

IL-17 is a proinflammatory cytokine produced by T cells, natural killer (NK) cells, and neutrophils that has an important role in the inflammatory responses [18,124]. Serum IL-17 presented a strong correlation with disease activity parameters in recent studies [124,125,126]. Also, Dedong et al. found that the serum levels of IL-17 can predict both clinical disease activity evaluated with SLEDAI score (AUC 0.91 to predict SLEDAI > 9) and histological disease activity (AUC 0.81 to predict AI > 8) [125]. Also, Saif et al. found a diagnostic role of IL-17 with an AUC of 0.92, Sn of 88.8% and Sp of 65% for differentiating LN patients from those with SLE without renal involvement; also, their group found an AUC of 0.86 for predicting the presence of a proliferative class of LN [127].

TWEAK is a cytokine member of the tumor necrosis factor superfamily that participates in inflammatory and fibrotic processes [128] and is a promising biomarker for the diagnosis and monitoring of LN. Results from different studies support the role of urinary TWEAK for the diagnosis of lupus nephritis (AUC 0.73–1.0, NPV 78.4–100%) [59,129,130,131,132,133] and for disease activity monitoring (AUC 0.96, Sn 100%, Sp 80%, OR 2.02) [129]. In the study conducted by Selim, only urinary TWEAK remained a significant independent predictor for renal flare in the multivariate analysis, with an OR of 2.02 (*p* = 0.03), whereas the complement fractions and anti-dsDNA were not statistically significant [134].

#### 3.4.2. Chemokines

Chemokines are soluble mediators of migration and recruitment in the kidney of infiltrating cells that express the corresponding chemokine receptor [18]. The most studied chemokines with promising results are represented by interferon-γ inducible protein-10 (IP-10) and monocyte chemoattractant protein-1 (MCP-1).

C-X-C motif ligand 10 (CXCL10), also known as IP-10, is a chemokine that is produced in response to IFN-γ activation and stimulates the migration of T lymphocytes to the inflammatory sites [135,136]. The potential role of urine CXCL10 as a diagnostic tool in LN patients was shown in the studies conducted by Kloche and Stanley (AUC 0.88–0.94, Sn 85.19–87.18%, Sp 80–100%) [137,138]. In the retrospective study conducted by Wang and Cui, serum levels of CXCL10 presented satisfactory ROC parameters for diagnosis of LN in SLE patients with an AUC of 0.75, Sn of 76.47% and Sp of 68.48%, but combining serum CXCL9, CXCL10 and CXCL11 demonstrated an improved receiver operating characteristic (ROC) profile with an AUC of 0.94, Sn 86.76 and Sp of 83.7% [139].

MCP-1, also referred to as C-C motif ligand 2, is a chemokine expressed by activated monocytes/macrophages, T cells, and NK cells and is one of the most studied novel biomarkers in LN [140]. Results from recent studies support the role of urinary MCP-1 for distinguishing LN from non-renal SLE (AUC 0.73–1.0, Sn 37.5–95%, Sp 58–97.3%, PPV 60–94%, NPV 68–95%) [59,87,104,131,133,137,141,142] and for identifying clinical (based on SLEDAI—AUC 0.7–1.0, Sn 70–100%, Sp 58–100%, PPV 100%, NPV 100%) and histological activity (for predicting proliferative LN- AUC 0.64–0.78; for predicting high AI-AUC 0.71, Sn 79%, Sp 65%) [45,46,68,104,133,143,144,145,146,147]. Moreover, Davies et al. suggested that the baseline levels of urinary MCP-1 can be an indicator of future response to therapy with Rituximab at 6 months (adjusted OR 2.6) and at 12 months (adjusted OR 0.6) either alone, or in combination with other urinary proteins including lipocalin-like prostaglandin D synthase, transferrin, alpha-1-acid glycoprotein, ceruloplasmin and the soluble isoform of the adhesion protein vascular cell adhesion molecule-1 (VCAM-1) with an AUC of 0.81 [148].

#### 3.4.3. Adhesion Molecules

Cellular adhesion molecules are essential for the interactions between immune cells involved in LN pathogenesis and the endothelium, and the guiding of leukocytes to the sites of inflammation [135,149]. Many cohorts have investigated the role of urinary activated leukocyte cell adhesion molecule (ALCAM) and VCAM-1 as predictors for the diagnosis and the disease activity of LN, managing to outperform the conventional biomarkers [149].

Elevated urinary levels of ALCAM demonstrated a good ability in discriminating patients with LN from non-renal SLE or inactive SLE (AUC 0.75–0.96, Sn 60–94%, Sp 53.3–100%, PPV 86–98%, NPV 65.5–92%) and healthy controls (AUC 0.82–0.98) [115,141,150,151,152,153]. Also, urinary ALCAM showed a potential role in distinguishing active from inactive LN (AUC 0.64–0.99, OR 1.52) [151,152]. Moreover, Ding et al. showed that urinary ALCAM outperformed conventional biomarkers in distinguishing proliferative LN from class V LN (AUC 0.81 for urinary ALCAM compared with AUC 0.58 for anti-dsDNA, AUC 0.77 for C3, AUC 0.58 for C4, and AUC 0.59 for proteinuria) [115]; also, it showed a good correlation with renal pathology AI (r = 0.40, *p* < 0.001) [115].

Also, urinary VCAM-1 has shown promise as a potential LN diagnosis biomarker (AUC 0.73–0.93, Sn 66.7–96%, Sp 65–100%, NPV 60–96.4%, PPV 61.5–95% vs. non-renal SLE/inactive SLE; AUC 0.77–0.98 vs. healthy controls) [59,88,141,142,147,150,154] and as a disease activity biomarker for predicting high SLEDAI scores or renal flares in several cohorts of SLE patients (SLEDAI- AUC 0.72–0.86, Sn 69–76%, Sp 73–90%; renal flares- AUC 0.76, Sn 75%, Sp 74%, HR 7.5) [88,96,147,150,154,155]. Furthermore, a combination panel of novel and traditional biomarkers composed of lipocalin-type prostaglandin D synthase L-(PGDS), intercellular adhesion molecule-1 (ICAM-1), VCAM-1, anti-dsDNA, C3, and C4 proved to have an AUC of 0.98 in predicting renal flares, outperforming a panel of only traditional biomarkers (C3, C4, and anti-dsDNA- AUC 0.88) in the exploratory study conducted by Fasano [96]. Also, high urinary VCAM-1 has been reported to correlate with organ damage markers and has the potential to identify the patients at increased risk for long-term renal function decline, defined as deterioration of eGFR by ≥25% at 10 years follow-up, with an AUC of 0.73 and an OR of 22.9 [143,156].

#### 3.4.4. Other Proteins

Cluster of differentiation 163 (CD163), a transmembrane protein and member of the cysteine-rich scavenger receptor family, is a marker of activated M2c macrophages, which are implicated in the pathogenesis of LN, being the predominant macrophage subtype found in LN biopsies [12,49,94,144,157]. As a diagnostic biomarker, recent studies have shown that urinary soluble CD163 has the following good ROC parameters (AUC 0.76–0.97, Sn 85%, Sp 100%, PPV 84.7%, NPV 100% vs. non-renal SLE/inactive SLE; AUC 0.90–0.97 vs. healthy controls) [86,94]. Moreover, it has been reported to correlate with LN disease activity in multiple, independent cohorts, being capable of differentiating the patients with active LN from SLE patients with inactive renal involvement (AUC 0.93–0.99, Sn 90.3–97%, Sp 88.89–100%, PPV 90.35–90.9%, NPV 88.9–100%) [6,89,158]. Also, correlations between the level of urinary soluble CD163 and AI have been reported [6,67,94,144,158,159], with some studies demonstrating that it is a reliable predictor in distinguishing a proliferative class of LN from a non-proliferative one with an AUC of 0.82–0.89 [68,94,144]. Also, urinary soluble CD163 may be used as an indicator of response to treatment [6,12,86,160], as suggested in one study conducted by Mejia-Vilet, where a urinary soluble CD163 value of <370 ng/mmol at month 6 predicted CRR at month 12 with a Sn of 90% and a Sp of 87%, outperforming conventional kidney disease-related and immuno-serological biomarkers [6]. Also, the same study suggested that a urinary soluble CD163 value of >370 ng/mmol translates into an increased probability of doubling of serum creatinine within 6 and 12 months with an HR of 2.82 and 3.62, respectively, raising the possibility of urinary soluble CD163 becoming also a reliable prognostic tool [6].

Neutrophil gelatinase-associated lipocalin (NGAL), also known as lipocalin-2, is a member of the lipocalin family that is involved in the proliferation of T cells, production and migration of cytokines, and regulation of immune inflammation [128,149]. Results from different cohorts support the role of urinary NGAL for discriminating LN patients from non-renal SLE patients (AUC 0.69–0.99, Sn 67–98%, Sp 48–100%, PPV 86.11%, NPV 71.43%) [59,104,161,162,163,164]. Also, urinary NGAL showed a significant correlation with renal SLEDAI and AI [143,161,164,165,166] and presented satisfactory ROC parameters for predicting response to therapy (AUC 0.724–0.943, Sn 72.7–92.31%, Sp 68.4–88.89%, PPV 55.6–90%, NPV 94.6–96%) in recent studies [70,143,167], outperforming traditional biomarkers (creatinine, eGFR, proteinuria) [70,167].

#### 3.4.5. Immune Cells

Interstitial, intra- and peri-glomerular inflammatory infiltration is an important feature of proliferative LN, and recent studies using single-cell transcriptomic analyses revealed the heterogeneous profile of the kidney-infiltrating immune cells [18,168]. Arazi et al. identified multiple populations of myeloid cells, T cells, NK cells, and B cells that are active in LN kidney samples, with a good correlation of gene expression between urine and kidney immune cells [168]. Recent studies suggested that urinary T cells are a promising tool for the diagnosis and assessment of LN. In the study conducted by Kopetschke, urinary CD8^+^ and CD4^+^ CD3^+^T cells correlated significantly with disease activity assessed by SLEDAI (r = 0.76, *p* < 0.0001 and r = 0. 73, *p* < 0.0001, respectively) and identified acute proliferative LN among SLE patients’ cohort, yielding very good ROC curve results (AUC 1, Sn 100%, Sp 100% and AUC 0.99, Sn 100%, Sp 98.25%, respectively), outperforming proteinuria (AUC 0.92) and serum creatinine (AUC 0.60) [44]. Also, Kim et al. found that urinary CD3^+^ T cells can distinguish proliferative LN from non-proliferative LN patients with an AUC of 0.82 [100].

#### 3.4.6. Microparticles

Microparticles (MPs), a subtype of extracellular vesicles, are released from apoptotic or activated cells in response to diverse stimuli. Recent studies showed a high number of MPs in SLE patients, most likely derived from abnormal cell activation and apoptosis, and consequently, dysfunctional clearance of the emerged MPs [169]. Lu et al. characterized the urinary podocyte-derived MPs and their association with LN, finding a potential diagnosis (AUC 0.96) and disease activity prediction role (AUC 0.78) of the urinary Annexin V^+^ podocalyxin^+^ MPs [169]. Another research group, conducted by Burbano, performed flow cytometry in urine and plasma samples to analyze the circulating monocytes and MPs in SLE patients with or without renal involvement [170]. Their group suggests that circulating MPs that carry alarmin-like elements on their surface, like high mobility group box 1 (HMGB1^+^), play a role in the activation of non-classical monocytes in SLE patients and their migration to the kidney tissue, followed by the release of more MPs from those activated monocytes, intensifying this pathogenic loop [170]. They found good ROC curve parameters for discriminating LN patients from non-renal SLE with an AUC of 0.84 for urinary MP-CX3CR1^+^, 0.96 for urinary MP-HLADR^+^, and 1 for urinary MP-HMGB1^+^. Moreover, urinary MP-HMGB1^+^ could predict the disease activity of LN with an AUC of 0.83 and a good Sp of 93.3% [170].

#### 3.4.7. MicroRNAs

Micro ribonucleic acids (miRNAs) are small non-coding ribonucleic acids (RNAs) that regulate messenger RNA by controlling its degradation and its translation into protein; they are transported in body fluids by forming complexes with proteins and high-density lipoproteins and by secretion in exosomes [171,172]. Quantitative reverse transcription polymerase chain reaction is the preferred method for detecting miRNA, being able to detect miRNA molecules at very low concentrations; the technique involves two steps: reverse transcription and amplification of the deoxyribonucleic acid by polymerase chain reaction [173]. The miRNA received special attention in SLE patients because of the indispensable roles of miRNA in regulating the development of immune cells and the innate and adaptive immune responses [18]. The last years have brought great progress in the research of miRNA as novel epigenetic biomarkers in LN, the most studied ones being miRNA-21, miRNA-146a and miRNA-155.

Serum miRNA-21 could play a role as a diagnostic biomarker in identifying LN patients (AUC 0.79–0.89, Sn 55%, Sp 100%, ORa 3.17 vs. non-renal SLE/inactive LN; AUC 0.912, Sn 86%, Sp 63%, PPV 76%, NPV 93% vs. healthy controls) [174,175,176,177]. Also, urinary exosomal miRNA-21 correlates with the histological chronicity index (CI_ (r = 0.565, *p* < 0.0001) [178]. Additionally, a combination of urinary exosomal miRNA-21, miRNA-150, and miRNA-29c showed very good results in predicting organ damage by distinguishing low CI from moderate-high CI with an AUC of 0.966, Sn of 94.4% and Sp of 99.8% [178].

Both serum and urine miRNA-146a displayed a good capability to distinguish LN patients from patients with non-renal SLE (serum-AUC 0.9–0.94, Sn 90–91.97%, Sp 80–100%, urine-AUC 0.81, Sn 67%, Sp 88%) or healthy controls (serum-AUC 0.75, Sn 56%, Sp 96%; urine-AUC 0.94, Sn 100%, Sp 83%) [174,179,180,181,182]. Moreover, baseline urinary exosomal miRNA-146a predicted the risk of future SLE flare at 36 months follow-up with an AUC of 0.89 and an OR of 7.08 (*p* = 0.02) [180].

Another promising biomarker for the diagnosis of LN is serum miRNA-155, showing an AUC of 0.70–0.85 when compared with non-renal SLE and an AUC of 0.82 when compared with healthy controls [175,177,181]. Zununi et al. showed that the combination panel composed of miRNA-125, miRNA-146, and miRNA-155 improved the performance of distinguishing LN patients from healthy controls, demonstrating an AUC of 0.89, Sn of 83% and Sp of 78% [181].

## 4. Discussion and Conclusions

The limitations of this review are represented by the absence of a systematic approach, the overall lack of external validation in independent cohorts, the heterogeneity of the studies included in terms of design, patient population, cut-off values, and definitions of outcomes, and the absence of a kidney biopsy for confirmation of LN in some of the studies. Also, the classification criteria applied for the diagnosis of SLE patients in the analyzed studies were either the 1982 or 1997 ACR, 2012 Systemic Lupus International Collaborating Clinics (SLICC), or 2019 EULAR/ACR. Furthermore, in some studies, the classification criteria used were not specified. The classification criteria used in the analyzed studies are mentioned for each study in Supplementary Table S1 [6,12,40,41,43,44,45,46,47,48,55,56,57,58,59,60,61,62,63,64,65,66,67,68,69,70,71,72,73,74,75,76,77,78,79,80,81,82,83,84,85,86,87,88,89,90,91,92,93,94,95,96,97,98,100,101,102,103,104,106,107,108,109,110,111,112,113,114,115,116,117,118,119,120,122,123,124,125,126,127,129,130,131,132,133,134,136,137,138,139,140,141,142,143,144,145,146,147,148,150,151,152,153,154,155,156,158,159,160,161,162,163,164,165,166,167,168,169,170,174,175,176,177,178,180,181,182].

Our search revealed that anti-dsDNA, serum C3, and proteinuria are the conventional biomarkers with the strongest clinical evidence, with overall moderate ability in predicting LN from non-renal SLE, disease activity, renal flares, response to therapy, and prognosis.

Several molecules, either alone or in combination panels, have been investigated over the last decade. Our search revealed promising results for urinary ALCAM, sCD163, CXCL10, MCP-1, NGAL, TWEAK, and VCAM-1. Urinary CD163 holds promise as a versatile biomarker for diagnosis, monitoring, prognosis, and prediction in LN that has survived independent validation cohorts. Also, ALCAM, MCP-1, NGAL, and VCAM-1 repeatedly showed promising potential for identifying LN in independent SLE cohorts. Next steps for clinical translation include establishing standardized assays and cut-off value definitions of these molecules and integration into current LN management.

Novel biomarkers should be validated in multiple longitudinal independent cohorts, compared with conventional biomarkers, and integrated with renal histology information, in order to optimize the management of LN patients, aiming for personalized precision medicine.

## Figures and Tables

**Table 1:** Definitions of complete renal response used in LN landmark randomized controlled trials.

Definitions of Complete Renal Response					References
Nomenclature	Proteinuria	Renal Function	Urinary Sediment	Additional Criteria	
Renal remission	Proteinuria < 1 g/day	Absence of a doubling of sCr level	<10 dysmorphic RBC/hpf and absence of cellular casts	-	Gourley et al. [22]
Complete remission	Proteinuria < 0.3 g/day	Values for sCr and CrCl no more than 15% above the baseline value	Normal urinary sediment	Normal serum albumin concentration	Chan et al. [23]
Complete response	Proteinuria < 1 g/day	sCr < 130% of the lowest level during treatment	<10 RBC/hpf and absence of cellular casts	Pt. had to be off IS therapy, with the exception of HQ (≤400 mg/day) and prednisone (≤10 mg/day) or their equivalents	Illei et al. [24]
Renal remission	Proteinuria < 1 g/day	Absence of a doubling of the sCr level	<10 RBC/hpf	-	Houssiau et al. [25]
Complete remission	Return to within 10% of normal values of proteinuria	Return to within 10% of normal values of sCr levels	Return to within 10% of normal values of urine sediment	-	Ginzler et al. [26]
Complete remission	Proteinuria ≤ 0.5 g/day	Return to normal sCr	Inactive urinary sediment (≤5 WBC/hpf and ≤5 RBC/hpf, and a reading of lower than 2+ on dipstick and absence of red cell casts)	-	Appel et al. [27]
Renal remission	Proteinuria < 1 g/day	sCr ≤ 1.4 mg/dL	<10 RBC/hpf	-	Houssiau et al. [28]
Complete renal remission	Proteinuria < 0.5 g/day	Improved or stable sCr ± 25% of baseline	Absence of hematuria and cellular casts	-	Dooley et al. [29]
Complete response	UPCR < 0.5	sCr within 10% of normal value	Normalized urine sediment	-	Zeher et al. [30]
Complete renal response	UPCR < 0.5	Normal sCr if it was abnormal at baseline or a sCr level of ≤115% of baseline if it was normal at baseline	Inactive urinary sediment (<5 RBCs/hpf and absence of RBC casts)	-	Rovin et al. [31]
Complete remission	UPCR < 50 mg/mmol	sCr no greater than 15% above baseline	-	-	Condon et al. [32]
Complete remission	Proteinuria ≤ 0.4 g/day	Normal sCr levels	Absence of active urine sediment	Serum albumin level of ≥35 g/L	Liu et al. [33]
Complete renal remission	UPCR ≤ 0.5 in 2 consecutive, first morning void urine specimens	eGFR > 60 mL/min/1.73 m^2^ or no decrease of ≥20% of baseline eGFR on 2 consecutive occasions	-	No administration of rescue medication and no more than 10 mg prednisone for more than 3 consecutive days or for more than 7 total days from weeks 16 to 26	Rovin et al. [34]
Primary efficacy renal response	UPCR ≤ 0.7	eGFR no worse than 20% below the pre-flare value or ≥60 mL/min/1.73 m^2^	-	No use of rescue therapy	Furie et al. [35]
Complete renal response	UPCR < 0.5	eGFR no worse than 10% below the pre-flare value or ≥90 mL/min/1.73 m^2^	-	No use of rescue therapy	Furie et al. [35]
Complete renal response	UPCR ≤ 0.5	Stable renal function defined as eGFR ≥ 60 m/min/1.73 m^2^ or no confirmed decrease from baseline in eGFR of >20%	-	No administration of rescue medication and no more than 10 mg prednisone equivalent per day for 3 or more consecutive days or for 7 or more days during weeks 44 through 52	Rovin et al. [36]
Complete renal response	UPCR < 0.5	Normal renal function (sCr ≤ ULN) without worsening of baseline sCr by more than 15%	Inactive urinary sediment (<10 RBC/hpf without RBC casts)	No administration of rescue therapies such as cyclophosphamide, rituximab, tacrolimus or pulse-dose corticosteroids (equivalent to methylprednisolone 500 mg or greater) after baseline	Furie et al. [37]
Complete renal response	UPCR ≤ 0.5	eGFR ≥ 60 m/min/1.73 m^2^ or no confirmed decrease from pretreatment baseline in eGFR of >20%	-	No administration of rescue medication and no more than 10 mg prednisone for ≥3 consecutive days or for ≥7 days in total during the eight weeks prior to the endpoint assessment	Saxena et al. [38]
Complete renal response	UPCR < 0.5	An eGFR of at least 85% of the baseline value	-	No administration of rescue therapy (except for glucocorticoid-only rescue)	Furie et al. [39]

CrCl: creatinine clearance; eGFR: estimated glomerular filtration rate; Hpf: high-power field; HQ: hydroxychloroquine; IS: immunosuppression; Pt.: patients; RBC: red blood cells; sCr: serum creatinine; ULN: upper limit of normal; UPCR: urine protein-creatinine ratio; WBC: white blood cells.

**Table 2:** The performances of kidney disease-related markers.

Biomarker	Sample	Comparator	Disease Activity Evaluation	Metrics and Findings	References
**Diagnosis**					
**Albumin-creatinine ratio**	Urine	Active non-renal SLE	NA	**AUC** 0.963	Häyry et al. [65]
**Creatinine**	Serum/plasma	Active non-renal SLE; Non-proliferative LN	NA	**AUC** 0.76, **Sn** 58%, **Sp** 96%	Landolt-Marticorena et al. [59]
	Serum/plasma	Non-renal SLE	NA	**AUC** 0.82, **Sn** 74.6%, **Sp** 76.1%, **PPV** 85.8%, **NPV** 60.7%	Yang et al. [60]
**Proteinuria**	Urine	Non-renal SLE; Inactive LN	NA	Proteinuria/24 h-**AUC** 0.92; **Sn** 93.75%, **Sp** 83.87%	Kopetschke et al. [44]
	Urine	Active non-renal SLE	NA	UPCR-**AUC** 0.93	Aggarwal et al. [43]
**Disease activity**					
**Albumin-creatinine ratio**	Urine	Non-proliferative LN	Proliferative LN	**AUC** 0.71	Häyry et al. [65]
	Urine	Inactive LN	SLEDAI	**AUC** 0.98, **Sn** 88.89%, **Sp** 100%	Wong et al. [61]
**Creatinine**	Serum/plasma	Inactive LN	SLEDAI	**AUC** 0.62, **Sn** 84.62%, **Sp** 35%	Wong et al. [61]
	Serum/plasma	Inactive LN	SLEDAI	**AUC** 0.68	Alharazy et al. [46]
**CystatinC**	Serum/plasma	Inactive LN	SLEDAI	**AUC** 0.90, **Sn** 75.7%, **Sp** 94.6%	Xu et al. [66]
**eGFR CystatinC**	Serum/plasma	Inactive LN	SLEDAI	**AUC** 0.90, **Sn** 75.7%, **Sp** 94.6%	Xu et al. [66]
**Granular casts**	Urine	Inactive LN	SLEDAI	**AUC** 0.91, **Sn** 82.4%, **Sp** 100%	Jakiela et al. [45]
**Proteinuria**	Urine	Inactive LN	SLEDAI	UPCR-**AUC** 0.94	Alharazy et al. [46]
	Urine	Inactive LN	AI	Proteinuria/24 h-AI > 2 AUC 0.69, Sn 48.5%, Sp 88.8%, PPV 94.1%, NPV 32%; AI > 3-AUC 0.75, Sn 50%, Sp 90%, PPV 94.1%. NPV 36%	Obrișcă et al. [67]
	Urine	Non-renal SLE	SLEDAI	Proteinuria/24 h-**AUC** 0.99, **Sn** 88.2%, **Sp** 100% (cut off >1.1 g/24 h), **Sn** 100%, **Sp** 90.9% (cut off >0.4 g/24 h)	Jakiela et al. [45]
	Urine	Non-proliferative LN	Proliferative LN	UPCR-**AUC** 0.74	Kitagawa et al. [68]
	Urine	Inactive LN	SLEDAI	UPCR-**AUC** 0.89	Gupta et al. [48]
**Red blood cells**	Urine	Non-renal SLE	SLEDAI	**AUC** 0.92, **Sn** 76.5%, **Sp** 100%	Jakiela et al. [45]
	Urine	Inactive LN	SLEDAI	**AUC** 0.72	Alharazy et al. [46]
	Urine	Inactive LN	AI	**AI > 9-AUC 0.7, Sn 77.7%, Sp 66.6%, PPV 63.6%, NPV 80%**	Obrișcă et al. [67]
	Urine	Class V LN	Proliferative LN	**OR 3.22**	Calatroni et al. [58]
**White blood cells**	Urine	Inactive LN	SLEDAI	**AUC** 0.75, **Sn** 70.6%, **Sp** 75%	Jakiela et al. [45]
	Urine	Inactive LN	SLEDAI	**AUC** 0.65	Alharazy et al. [46]
**Renal flares**					
**Creatinine**	Serum/plasma	NA	NA	sCr at presentation—**HR** 1.76	Mejía-Vilet et al. [69]
**Proteinuria**	Urine	NA	NA	Proteinuria (g/24 h) at first visit and LN flare—**HR** 1.004; Proteinuria > 500 mg/24 h and LN flare—**NPV** 85%, **PPV** 43%	Fatemi et al. [47]
	Urine	NA	NA	Proteinuria > 0.8 g/24 h at 12 months- a higher risk of flares (**OR** 4.12) Baseline proteinuria > 2 g/24 h and 12 months proteinuria > 0.8 g/24 h- a shorter time to flare (**HR** 2.56 and **HR** 2.57)	Kapsia et al. [55]
**Response to therapy**					
**Creatinine**	Serum/plasma	NA	NA	Baseline sCr to predict CRR-**AUC** 0.85, **Sn** 76.92%, **Sp** 92.59%, **PPV** 83.3%, **NPV** 89.3%	El-Mohsen et al. [70]
**Proteinuria**	Urine	NA	NA	The changes in UPCR at 3 months predict 1-year response- **AUC** 0.76 in proliferative LN	Fava et al. [12]
	Urine	NA	NA	Baseline 24 h proteinuria-**OR** 0.63: negative predictor of improvement and CRR at 6 months	McDonald et al. [71]
	Urine	NA	NA	Baseline levels 0.1–0.87 g/24-predictive of CRR at 6 months (**OR** 4.3)	Ichinose et al. [56]
	Urine	NA	NA	UPCR (<1.5 g/g at month 6)-**Sn** 86%, **Sp** 81%, **PPV** 81%, **NPV** 86% to predict CRR by month 12 25% UPCR reduction at month 6-**Sn** 86%, **Sp** 65%, **PPV** 69%, **NPV** 83% to predict CCR by month 12	Mejia-Vilet et al. [6]
	Urine	NA	NA	Baseline UPCR-**AUC** 0.80, **Sn** 76.92%, **Sp** 88.89%, **PPV** 80%, **NPV** 96%	El-Mohsen et al. [70]
**Prognosis**					
**Creatinine**	Serum/plasma	NA	NA	Higher baseline levels are predictive of **ESKD-HR** 2.1	Chen et al. [64]
	Serum/plasma	NA	NA	**Good long-term renal outcome = sCr ≤ 1 mg/dL after 7 years** sCr ≤ 0.8 mg/dL at 12 months-**Sn** 58%, **Sp** 83%, **PPV** 88%, **NPV** 49% sCr ≤ 1 mg/dL at 12 months-**Sn** 90%, **Sp** 48%, **PPV** 78%, **NPV** 69%	Dall’Era et al. [40]
	Serum/plasma	NA	NA	**Risk of sustained 30% decline in eGFR**-baseline sCr: **HR** 1.19; **sustained 50% decline in eGFR**—**HR** 1.23**, ESKD**-**HR** 1.24	Whittal Garcia et al. [63]
	Serum/plasma	NA	NA	**Risk factor for death and doubling of sCr/ESKD**-HRa 4.65	Pang et al. [62]
**eGFR Creatinine**	Serum/plasma	NA	NA	<60 mL/min/1.73 m^2^ at the onset of LN-**predictor of development of CKD**: **HR** 4.91	Park et al. [72]
**Proteinuria**	Urine	NA	NA	**Good long-term renal outcome = sCr ≤ 1 mg/dL after 7 years** Proteinuria < 0.8 g/24 h at 12 months **Sn** 81%, **Sp** 78%, **PPV** 88%, **NPV** 67%	Dall’Era et al. [40]
	Urine	NA	NA	Baseline UPCR > 3.1 g/Cr-**AUC** 0.64, **Sp** 41.5%, **Sn** 80%-**predicted decreased eGFR at 12 months**	Nozaki et al. [73]
	Urine	NA	NA	Proteinuria (g/24 h) at diagnosis**-predictive of renal failure (dialysis or KT)** within 20 years-**RRa** 2.75	Petri et al. [57]
	Urine	NA	NA	**RR of incident ESKD** 0.11**, RR of composite outcome (sum of mortality and incidence of ESKD**) 0.21	Koo et al. [74]
	Urine	NA	NA	12-month proteinuria > 0.8 g/24 h—**OR** 10.8 for **CKD stage 3–4/ESKD**	Kapsia et al. [55]
	Urine	NA	NA	UPCR at 12 months t**o predict long-term renal outcome (doubling of sCr/sCr > 4 mg/dL (if initial Cr > 2.5 mg/dL)/ESKD** at 48 months)-**AUC** 0.65**, Sn** 77%, **Sp** 66%, **PPV** 36%, **NPV** 91%, **HR** 1.45	Domingues et al. [75]
	Urine	NA	NA	Proteinuria at 12 months < 0.7 g/24 h to predict **good long-term renal outcome (sCr ≤ 1 mg/dL after 7 years)-Sn** 71%, **Sp** 75%, **PPV** 94%, **NPV** 31%	Tamirou et al. [41]
**Red blood cells**	Urine	NA	NA	RBC ≤ 5/hpf at 12 months to predict **good long-term renal outcome = sCr ≤ 1 mg/dL after 7 years**: **Sn** 62%, **Sp** 64%, **PPV** 78%, **NPV** 45%	Dall’Era et al. [40]

AI: activity index; AUC: area under the curve; CKD: chronic kidney disease; CRR: complete renal response; eGFR: estimated glomerular filtration rate; ESKD: end-stage kidney disease; HC: healthy controls; HR(a): (adjusted) hazard ratio; KT: kidney transplant; LN: lupus nephritis; NPV: negative predictive value; NA: not applicable; OR(a): (adjusted) odds ratio; PCR: protein-creatinine ratio; PPV: positive predictive value; pt.: patients; RBC: red blood cells; RR(a): adjusted relative risk; sCr: serum creatinine; SLE: systemic lupus erythematosus; SLEDAI: Systemic Lupus Erythematosus Activity Index; Sn: sensitivity; Sp: specificity; UPCR: urine protein-creatinine ratio. 24 h: 24 hours.

**Table 3:** The performance of serum antibodies.

Biomarker	Sample	Comparator	Disease Activity Evaluation	Metrics and Findings	References
**Diagnosis**					
**Anti-C1q**	Serum/plasma	Non-renal SLE	NA	**AUC** 0.753	Renaudineau et al. [89]
	Serum/plasma	Non-renal SLE	NA	**Sn** 53%, **Sp** 85%, **NPV** 67%, **PPV** 76%	Plawecki et al. [81]
	Serum/plasma	Non-renal SLE	NA	**AUC** 0.843, **Sn** 68.75%, **Sp** 84%; **OR** 14.79	Gargiulo et al. [82]
	Serum/plasma	Non-renal SLE	NA	**Sn** 53%, **Sp** 87%, **NPV** 87%, **PPV** 61%	Colliard et al. [101]
	Serum/plasma	Non-renal SLE	NA	**OR** 4.4	Sjöwall et al. [83]
	Serum/plasma	Active non-renal SLE	NA	**AUC** 0.64, **Sn** 47%, **Sp** 83%	Pang et al. [62]
	Serum/plasma	Non-renal SLE	NA	**Sn** 63%, **Sp** 71%	Birmingham et al. [102]
	Serum/plasma	Non-renal SLE	NA	**Sn** 85.7%, **Sp** 66.7%, **PPV** 60%, **NPV** 88.9%, **OR** 12	Chi et al. [103]
	Serum/plasma	Non-renal SLE	NA	**AUC** 0.76, **Sn** 74%, **Sp** 55%	Gómez-Puerta et al. [104]
	Serum/plasma	Non-renal SLE	NA	**Sn** 59%, **Sp** 63%, **PPV** 64%, **NPV** 58%, **OR** 2.40	Jia et al. [105]
**Anti-dsDNA**	Serum/plasma	Non-renal SLE	NA	**AUC** 0.72, **Sn** 72%, **Sp** 72.33%, **HR** 5.84, **HRa** 2.67	Liu et al. [76]
	Serum/plasma	Non-renal SLE	NA	**AUC** 0.89, **Sn** 100%, **Sp** 71%, **PPV** 44%, **NPV** 100%, **HR** 1.06	Kwon et al. [77]
	Serum/plasma	Active non-renal SLE; inactive SLE	NA	**Sn** 94%, **Sp** 40%, **PPV** 43%, **NPV** 93%	Mok et al. [78]
	Serum/plasma	Non-renal SLE	NA	**OR** 2.1	Hardt et al. [79]
	Serum/Plasma	Non-renal SLE	NA	**OR** 3.27	Barnado et al. [80]
	Serum/plasma	Non-renal SLE	NA	**Sn** 92%, **Sp** 32%, **NPV** 81%, **PPV** 54%	Plawecki et al. [81]
	Serum/plasma	Non-renal SLE	NA	**AUC** 0.70, **Sn** 56.25%, **Sp** 88%, **OR** 9.43	Gargiulo et al. [82]
	Serum/plasma	non-renal SLE	NA	**OR** 2.9	Sjöwall et al. [83]
	Serum/plasma	Non-renal SLE	NA	**HRa** 1.004	Kwon et al. [84]
	Serum/plasma	Non-renal SLE; HC	NA	Non-renal SLE-**AUC** 0.65; HC-**AUC** 0.94	Bruschi et al. [85]
	Serum/plasma	Active non-renal SLE	NA	**AUC** 0.61	Aggarwal et al. [43]
	Serum/plasma	Active non-renal SLE; inactive SLE	NA	**AUC** 0.6	Gupta et al. [86]
	Serum/plasma	Active non-renal SLE	NA	**AUC** 0.64, **Sn** 63%, **Sp** 64%	Mok et al. [88]
	Serum/plasma	Active non-renal SLE	NA	**AUC** 0.72, **Sn** 71.43%, **Sp** 70.37%	Wong et al. [61]
	Serum/plasma	Non-renal SLE	NA	**AUC** 0.79	Renaudineau et al. [89]
**Anti-Sm**	Serum/plasma	Non-renal SLE	NA	**HRa** 2.09	Kwon et al. [84]
	Serum/plasma	Non-renal SLE	NA	**Sn** 74%, **Sp** 83%, **PPV** 93%, **NPV** 53%-diagnosis of silent LN	Ishizaki et al. [107]
**Disease activity**					
**Anti-C1q**	Serum/plasma	Isolated class V LN	Proliferative LN	**AUC** 0.73, **Sp** 47.7%, **Sn** 100%	Renaudineau et al. [89]
	Serum/plasma	Non-proliferative LN	Proliferative LN, AI	**AUC** 0.71, **OR** 1.49; AI (**r** 0.27)	Fava et al. [90]
	Serum/plasma	Inactive LN	SLEDAI	**AUC** 0.73, **Sn** 62.9%, **Sp** 75%, **PPV** 69%, **NPV** 71%, **OR** 5.1	Kianmehr et al. [92]
	Serum/plasma	Inactive LN	SLEDAI	**OR** 8.4	Sjöwall et al. [83]
	Serum/plasma	N/A	SLEDAI, ECLAM	SLEDAI **(r** 0.47), ECLAM **(r** 0.28)	Bock et al. [108]
	Serum/plasma	Non-proliferative LN	Proliferative LN, AI	anti-dsDNA/anti-C1q-**OR** 8.67; AI **(r** 0.24)	Moroni et al. [93]
	Serum/plasma	Non-A BILAG category	BILAG, Class IV LN, AI	**AUC** 0.72, **Sn** 50%, **Sp** 74.2%; BILAG (**r** 0.34); Class IV LN-**Sn** 40%, **Sp** 93.5%, **PPV** 76.9%, **NPV** 74.1%, **OR** 9.5, AI (**r** 0.43)	Radanova et al. [106]
	Serum/plasma	Inactive LN	SLEDAI	**AUC** 0.76, **Sn** 72%, **Sp** 55%; SLEDAI (**r** 0.46)	Gómez-Puerta et al. [104]
	Serum/plasma	Class V LN	Proliferative LN	**OR** 1.02	Calatroni et al. [58]
**Anti-dsDNA**	Serum/plasma	Non-proliferative LN	Proliferative LN, AI	**AUC** 0.73, **OR** 1.37, AI **(r** 0.34)	Fava et al. [90]
	Serum/plasma	Class V LN	Proliferative LN ± Class V LN	**AUC** 0.83	Li et al. [95]
	Serum/plasma	Non-BILAG A	BILAG	**AUC** 0.66, **OR** 3.86	Vasilev et al. [91]
	Serum/plasma	Inactive LN	SLEDAI	**AUC** 0.88, **Sn** 70.6%, **Sp** 87.5%	Jakiela et al. [45]
	Serum/plasma	Inactive LN	SLEDAI	**AUC** 0.7, **Sn** 71.4%, **Sp** 62.5%, **PPV** 62.5%, **NPV** 67%; **OR** 4.2; (**r** 0.42)	Kianmehr et al. [92]
	Serum/plasma	Inactive LN	SLEDAI	**OR** 4.8	Sjöwall et al. [83]
	Serum/plasma	Non-proliferativeLN	Proliferative LN	**AUC** 0.72, **Sn** 82%, **Sp** 59%	Landolt-Marticorena et al. [59]
	Serum/plasma	Non-proliferative LN	Proliferative LN, AI	anti-dsDNA/antiC1q-**OR** 8.67; AI (**r** 0.31)	Moroni et al. [93]
	Serum/plasma	Non-proliferative LN	Proliferative LN	**AUC** 0.7	Zhang et al. [94]
	Serum/plasma	Inactive LN	SLEDAI	**AUC** 0.75, **Sn** 65%, **Sp** 65%	Mok et al. [88]
	Serum/plasma	Inactive LN	SLEDAI	**AUC** 0.68, **Sn** 73.08%, **Sp** 55.56%	Wong et al. [61]
	Serum/plasma	Isolated class V LN	Proliferative LN	**AUC** 0.81, **Sp** 44.4%, **Sn** 100%	Renaudineau et al. [89]
**Anti-Sm**	Serum/plasma	Isolated class V LN	Proliferative LN	**AUC** 0.72, **Sp** 44.4%, **Sn** 100%	Renaudineau et al. [89]
**Renal flares**					
**Anti-C1q**	Serum/plasma	NA	NA	**Sn** 70%, **Sp** 44%	Birmingham et al. [102]
	Serum/plasma	NA	NA	**NPV** 93%, **PPV** 35%; **HR** 1.009	Fatemi et al. [47]
	Serum/plasma	NA	NA	**Sn 67.6%, Sp 79.7%, PPV 62.5%, NPV 83.1%**	Vigne et al. [109]
Anti-C1s	Serum/plasma	NA	NA	**Sn 59.5%, Sp 81.1%, PPV 61.1%, NPV 80%**	Vigne et al. [109]
**Anti-dsDNA**	Serum/plasma	NA	NA	**Sn** 40%, **Sp** 90%, **PPV** 80%, **NPV** 60%	Himbert et al. [97]
	Serum/plasma SLE-ELISpot	NA	NA	High SLE-ELISpot: **HR** 6.5	Pérez-Isidro et al. [98]
	Serum/plasma	NA	NA	**AUC** 0.85, **Sn** 87%, **Sp** 83%, **PPV** 43%, **NPV** 97%, **HR** 21.67	Fasano et al. [96]
	Serum/plasma	NA	NA	**Sn 91.9%, Sp 36.5%, PPV 42%, NPV 90%**	Vigne et al. [109]
**Response to therapy**					
**Anti-dsDNA**	Serum/plasma	NA	NA	Disappearance at 6 months to predict a **CRR** at 12 months—**Sn** 70%, **Sp** 56%, **PPV** 67%, **NPV** 59% 25% reduction at 6 months to predict a **CRR** at 12 months**—Sn** 83%, **Sp** 45%, **PPV** 59%, **NPV** 74%	Mejia-Vilet et al. [6]
	Serum/plasma	NA	NA	Baseline anti-dsDNA+ inversely predicted **NRR** at 6 months-**OR** 0.32	Zhao et al. [110]
	Serum/plasma	NA	NA	AUC 0.73 to predict **NRR** at 6 months after treatment	Kim et al. [100]
**Prognosis**					
**Anti-C1q**	Serum/plasma			RF for **death and doubling of sCr/ESKD**-**HR** 3.9; **HRa** 1.2	Pang et al. [62]
**Anti-dsDNA**	Serum/Plasma			**Renal failure: OR** 2.3, **ESKD: OR** 2.53	Barnado et al. [80]
	Serum/plasma			Risk of **sustained 30% decline in eGFR**-**HR** 1.73; **sustained 50% decline in eGFR**-**HR** 1.97; **ESKD-HR** 1.89 Adverse renal event (**the time to the second renal flare and/or the time to at least a 30% sustained decline in eGFR**)-**AUC** 0.69**, Sn** 64%, **Sp** 62%, **PPV** 67%, **NPV** 59%, **HR** 1.62	Whittal-Garcia et al. [63]

AI: activity index; Anti-dsDNA: anti-double stranded deoxyribonucleic acid; AUC: area under the curve; BILAG: the British Isles Lupus Assessment Group; CKD: chronic kidney disease; CRR: complete renal response; ECLAM: European Consensus lupus activity measurement; eGFR: estimated glomerular filtration rate; HC: healthy controls; ESKD: end-stage kidney disease; HR(a): (adjusted) hazard ratio; LN: lupus nephritis; KT: kidney transplant; NPV: negative predictive value; NA: applied; NRR: no renal response; OR(a): (adjusted) odds ratio; PPV: positive predictive value; pt.: patients; RR(a): adjusted relative risk; sCr: serum creatinine; SLE: systemic lupus erythematosus; SLEDAI: Systemic Lupus Erythematosus Activity Index; SLE-ELISpot: anti-dsDNA autoantibodies secreting cells enzyme-linked immune sorbent spot; Sn: sensitivity; Sp: specificity.

**Table 4:** The performances of complement tests.

Biomarker	Sample	Comparator	Disease Activity Evaluation	Metrics and Findings	References
**Diagnosis**					
**C3**	Serum/plasma	Active non-renal SLE; inactive SLE	NA	**Sn** 97%, **Sp** 32%, **PPV** 41%, **NPV** 95%	Mok et al. [78]
	Serum/plasma	Active non-renal SLE	NA	**AUC** 0.66, **Sn** 66%, **Sp** 61%	Mok et al. [88]
	Serum/plasma	Non-renal SLE	NA	**AUC** 0.70	Renaudineau et al. [89]
	Serum/plasma	Non-renal SLE	NA	**HRa** 0.97	Kwon et al. [84]
	Serum/plasma	Active non-renal SLE	NA	**AUC** 0.68	Aggarwal et al. [43]
	Serum/plasma	Active non-renal SLE; Inactive SLE	NA	**AUC** 0.65	Gupta et al. [87]
	Serum/plasma	Active non-renal SLE	NA	**AUC** 0.91, **Sn** 85.71%, **Sp** 86.84%	Wong et al. [61]
	Serum/plasma	Active non-renal SLE	NA	**AUC** 0.78, **Sn** 74%, **Sp** 64%, **PPV** 67%, **NPV** 71%, **OR** 5.03	Martin et al. [111]
	Serum/plasma	Non-renal SLE	NA	**Sn** 78%, **Sp** 92%, **PPV** 97%, **NPV** 58%, **OR** 39-diagnosis of silent LN	Ishizaki et al. [107]
	Serum/plasma	Non-renal SLE	NA	**HR** 6.4	Liu et al. [76]
	Serum/Plasma	Activenon- renal SLE	NA	**AUC** 0.81	Phatak et al. [112]
**C4**	Serum/Plasma	Active non-renal SLE	NA	**AUC** 0.61	Phatak et al. [112]
	Serum/plasma	Active non-renal SLE; Inactive SLE	NA	**AUC** 0.62	Gupta et al. [87]
	Serum/plasma	Non-renal SLE	NA	**HR** 4.99	Liu et al. [76]
	Serum/plasma	Active non-renal SLE	NA	**AUC** 0.66	Aggarwal et al. [43]
	Serum/plasma	Active non-renal SLE; Inactive SLE	NA	**AUC** 0.62	Gupta et al. [86]
	Serum/plasma	Active non-renal SLE	NA	**AUC** 0.71, **Sn** 70%, **Sp** 68%, **PPV** 69%, **NPV** 70%; **OR** 5.1	Martin et al. [111]
**Disease activity**					
**C3**	Serum/plasma	Non-renal SLE; Inactive LN	AI	AI > 8-**AUC** 0.68	Liang et al. [116]
	Serum/plasma	Inactive LN	SLEDAI	**AUC** 0.82, **Sn** 74%, **Sp** 73%	Mok et al. [88]
	Serum/plasma	Inactive LN	SLEDAI	**AUC** 0.75	Gupta et al. [48]
	Serum/plasma	Non-proliferative LN	Proliferative LN	**AUC** 0.7	Zhang et al. [94]
	Serum/plasma	Inactive LN	SLEDAI	**AUC** 0.82	Ganguly et al. [113]
	Serum/plasma	Class V LN	Proliferative LN	**AUC** 0.77, **Sn** 75%, **Sp** 74%, **PPV** 92%, **NPV** 44%	Ding et al. [115]
	Serum/plasma	Class V LN	Proliferative LN ± class V LN	**AUC** 0.76	Li et al. [95]
	Serum/plasma	Inactive LN	SLEDAI	**AUC** 0.89, **Sn** 74.36%, **Sp** 84.21%	Wong et al. [61]
	Serum/plasma	N/A	SLEDAI	SLEDAI (**r**-0.99)	Selvaraja et al. [114]
	Serum/plasma	Inactive LN	SLEDAI	**AUC** 0.88, **Sn** 100%, **Sp** 64.7%	Jakiela et al. [45]
**C4**	Serum/plasma	Inactive LN	SLEDAI	**AUC** 0.68	Gupta et al. [48]
	Serum/plasma	Class V LN	Proliferative LN ± class V LN	**AUC** 0.68	Li et al. [95]
	Serum/plasma	Inactive LN	SLEDAI	**AUC** 0.87	Ganguly et al. [113]
	Serum/plasma	N/A	SLEDAI	SLEDAI (**r**-0.83)	Selvaraja et al. [114]
	Serum/plasma	Inactive LN	SLEDAI	**AUC** 0.88, **Sn** 81.3%, **Sp** 88.2%	Jakiela et al. [45]
**C4d**	Serum/plasma	N/A	AI	AI (**r** 0.37)	Martin et al. [111]
**Renal flares**					
**C3**	Serum/plasma	NA	NA	**Sn** 70%, **Sp** 59%, **OR** 2.5	Ruchakorn et al. [117]
	Serum/plasma	NA	NA	**AUC** 0.76, **Sn** 100%, **Sp** 50.9%, **PPV** 23%, **NPV** 100%, **HR** 5.95	Fasano et al. [96]
**C4**	Serum/plasma	NA	NA	**AUC** 0.82, **Sn** 100%, **Sp** 62.3%, **PPV** 28%, **NPV** 100%, **HR** 5.51	Fasano et al. [96]
	Serum/plasma	NA	NA	**ORa** 5.6	Buyon et al. [118]
**Response to therapy**					
**C3**	Serum/plasma	NA	NA	AUC 0.84 to predict **NRR** at 6 months after treatment	Kim et al. [100]
	Serum/plasma	NA	NA	C3 normalization at month 6**-Sn** 76%, **Sp** 55%, **PPV** 61%, **NPV** 71% to predict **CRR** by month 12 25% C3 increase at month 6-**Sn** 76%, **Sp** 68%, **PPV** 69%, **NPV** 75% to predict **CCR** by month 12	Mejia-Vilet et al. [6]
**C4**	Serum/plasma	NA	NA	**AUC** 0.65 to predict **NRR** at 6 months after treatment	Kim et al. [100]
**Prognosis**					
**C3**	Serum/plasma	NA	NA	Persistent isolated low C3 6 months after KB-**composite outcome of ESKD or death-HR** 2.46; **ESKD-HRa** 3.41	Rossi et al. [119]
	Serum/plasma	**NA**	NA	Low C3 ever-predictive of **renal failure (dialysis or KT)** within 20 years-**RRa** 2.0	Petri et al. [57]

AI: activity index; AUC: area under the curve; BILAG: the British Isles Lupus Assessment Group; CRR: complete renal response; eGFR: estimated glomerular filtration rate; ESKD: end-stage kidney disease; HC: healthy controls; HR(a): (adjusted) hazard ratio; LN: lupus nephritis; KT: kidney transplant; NPV: negative predictive value; NA: not applied; NRR: no renal response; OR(a): (adjusted) odds ratio; PPV: positive predictive value; pt.: patients; RR(a): adjusted relative risk; sCr: serum creatinine; SLE: systemic lupus erythematosus; SLEDAI: Systemic Lupus Erythematosus Activity Index; Sn: sensitivity; Sp: specificity.

## Data Availability

Not applicable.

## Supplementary Materials

The following supporting information can be downloaded at: https://www.mdpi.com/article/10.3390/life15101497/s1, Table S1: SLE Classification criteria used in the analysed studies.

## Abbreviations

The following abbreviations are used in this manuscript:

AI	activity index
ALCAM	activated leukocyte cell adhesion molecule
Anti-dsDNA	anti-double-stranded deoxyribonucleic acid
ACR	American College of Rheumatology
AUC	area under the curve
BAFF	B cell activating factor
BILAG	British Isles Lupus Assessment Group
BLyS	B lymphocyte stimulator
CD163	cluster of differentiation 163
CRR	complete renal response
CXCL	C-X-C motif ligand
DORIS	definitions of remission in SLE
DNA	deoxyribonucleic acid
eGFR	estimated glomerular filtration rate
ESKD	end-stage kidney disease
EULAR	European League Against Rheumatism
HR	hazard ratio
HMGB1^+^	high mobility group box 1
IL	interleukin
IP-10	interferon-γ inducible protein-10
KDIGO	kidney disease improving global outcomes
LN	lupus nephritis
MCP-1	monocyte chemoattractant protein-1
miRNAs	micro ribonucleic acids
MPs	microparticles
NGAL	neutrophil gelatinase-associated lipocalin
NK	natural killer
NPV	negative predictive values
OR	odds ratio
PCR	protein-creatinine ratio
PERR	primary efficacy renal response
PPV	positive predictive value
RNA	ribonucleic acid
ROC	receiver operating characteristic
SLE	systemic lupus erythematosus
SLEDAI	SLE disease activity index
SLE-ELISpot	anti-dsDNA autoantibodies secreting cells enzyme-linked immune sorbent spot
Sn	sensitivity
Sp	specificity
TWEAK	tumor necrosis factor-like weak inducer of apoptosis
VCAM-1	vascular cell adhesion molecule-1
