# Supplementary material for: Biomarkers in Lupus Nephritis: An Evidence-Based Comprehensive Review
Source: Life (Basel). 2025 Sep 23;15(10):1497. doi: 10.3390/life15101497 (PMC12565261; doi:10.3390/life15101497)
Supplement: Supplementary file 1 [file life-15-01497-s001.zip › life-3874475-supplementary.pdf]

Supplementary Table S1. SLE Classification criteria used in the analysed studies

Study	Classification criteria for SLE
Mejia-Vilet et al [1]	1997 ACR
Fava et al [2]	1997 ACR
Dall'Era et al[3]	1982 ACR
Tamirou et al [4]	1982 ACR
Aggarwal et al [5]	1997 ACR
Kopetschke et al [6]	NS
Jakiela et al [7]	1997 ACR
Alharazy et al [8]	1997 ACR
Fatemi et al [9]	1997 ACR
Gupta et al [10]	1982 ACR
.Kapsia et al [11]	2019 EULAR/ACR
Ichinose et al [12]	NS
Petri et al [13]	1997 ACR or 2012 SLICC
Calatroni et al[14]	1997 ACR
Landolt-Marticorena et al [15]	1997 ACR
Yang et al [16]	1997 ACR
Wong et al [17]	NS
Pang et al [18]	1997 ACR
Whittall Garcia et al [19]	2019 EULAR/ACR
Chen et al [20]	1997 ACR
Häyry et al [21]	1982 ACR or 2012 SLICC
Xu et al [22]	1997 ACR
Obrișcă et al [23]	NS
Kitagawa et al [24]	NS
Mejia-Vilet et al[25]	NS
El-Mohsen et al [26]	1982 ACR
McDonald et al [27]	1997ACR
Park et al [28]	1997 ACR
Nozaki et al [29]	NS
Koo et al [30]	ACR
Domingues et al [31]	1982 ACR or 2012 SLICC
Liu et al [32]	1997 ACR
Kwon et al [33]	1997 ACR
Mok et al [34]	1997 ACR
Hardt et al [35]	1982 ACR
Barnado et al [36]	1997 ACR
Plawecki et al [37]	1997 ACR
Gargiulo et al [38]	1997 ACR
Sjöwall et al [39]	1997 ACR
Kwon et al [40]	1997 ACR
Bruschi et al [41]	2012 SLICC

Gupta et al [42]	1997 ACR
Gupta et al [43]	1997 ACR
Mok et al [44]	1997 ACR
Renaudineau et al [45]	2019 EULAR/ACR
Fava et al [46]	1997 ACR or 2012 SLICC
Vasilev et al [47]	ACR
Kianmehr et al [48]	1997 ACR
Moroni et al [49]	1997 ACR
Zhang et al[50]	2012 SLICC
Li et al [51]	NS
Radanova et al [52]	ACR
Fasano et al [53]	1997 ACR
Himbert et al [54]	1997 ACR
Pérez-Isidro et al [55]	2019 EULAR/ACR
Kim et al [56]	1997 ACR
Colliard et al [57]	1997 ACR
Birmingham et al [58]	ACR
Chi et al [59]	1982 ACR
Gómez-Puerta et al [60]	1982/1997 ACR
Jia et al [61]	1997 ACR
Ishizaki et al [62]	1997 ACR
Bock et al [63]	1997 ACR
Vigne et al [64]	1997 ACR
Zhao et al [65]	1997 ACR or 2012 SLICC
Martin et al [66]	1982 ACR or 2012 SLICC
Phatak et al [67]	2012 SLICC
Ganguly et al[68]	2012 SLICC
Selvaraja et al [69]	ACR
Ding et al [70]	1997 ACR or 2012 SLICC
Liang et al [71]	1997 ACR
.Ruchakorn et al [72]	1997 ACR or 2012 SLICC
Buyon et al [73]	1997 ACR
Rossi et al [74]	2019 EULAR/ACR
Parodis et al [75]	1982 ACR and 2012 SLICC
Li et al [76]	NS
Vincent et al [77]	1997 ACR
Abdel Galil et al [78]	1997 ACR
Dedong et al [79]	1997 ACR
Nordin et al [80]	1982 ACR or 2012 SLICC
Saif et al [81]	2012 SLICC
Salem et al [82]	1997 ACR
Susianti et al [83]	2012 SLICC
Dong et al [84]	1997 ACR
Reyes-Martínez et al [85]	2012 SLICC
Elsaid et al [86]	2012 SLICC
Selim et al [87]	2012 SLICC

Abediazar et al [88]	NS
Klocke et al [89]	NS
Stanley et al [90]	NS
Wang et al [91]	1997 ACR
Dong et al [92]	1997 ACR
Stanley et al [93]	NS
Whittall-Garcia et al [94]	1997 ACR
Liu et al [95]	1997 ACR
Endo et al [96]	NS
Bona et al [97]	2012 SLICC
Ngamjanyaporn et al [98]	1997 ACR
Lai et al [99]	1997 ACR
Davies et al [100]	NS
Soliman et al [101]	1997 ACR
Chalmers et al [102]	2012 SLICC
Amer et al [103]	2019 EULAR/ACR
Kim et al [104]	2012 SLICC or 2019 EULAR/ACR
Yu et al [105]	NS
Gasparin et al [106]	1997 ACR
Parodis et al[107]	1982 ACR and 2012 SLICC
Gamal et al [108]	2012 SLICC
Yang et al [109]	1997 ACR
Fava et al [110]	NS
Susianti et al [111]	1997 ACR
Li et al [112]	1997 ACR
Ibrahim et al [113]	1997 ACR
El Shahawy et al [114]	1997 ACR
Susianti et al [115]	ACR
Elewa et al [116]	1997 ACR
Satirapoj et al[117]	1982 ACR
Arazi et al [118]	NS
Lu et al [119]	ACR
Burbano et al [120]	1997 ACR
ElFeky et al [121]	2019 EULAR/ACR
Khoshmirsafa et al [122]	1997 ACR
Nakhjavani et al [123]	NS
Li et al [124]	1997 ACR
Solé et al [125]	1997 ACR
Perez-Hernandez et al [126]	NS
Zununi et al [127]	ACR
Higazi et al [128]	2012 SLICC

ACR: American College of Rheumatology; EULAR: European League Against Rheumatism; NS: not specified; SLE: systemic lupus erythematosus; SLICC: Systemic Lupus Erythematosus International Collaborating Clinics.

References

1. Mejia-Vilet, J.M.; Zhang, X.L.; Cruz, C.; Cano-Verduzco, M.L.; Shapiro, J.P.; Nagaraja, H.N.; Morales-Buenrostro, L.E.; Rovin, B.H. Urinary Soluble CD163: A Novel Noninvasive Biomarker of Activity for Lupus Nephritis. *J. Am. Soc. Nephrol.* **2020**, *31*, 1335–1347. https://doi.org/10.1681/ASN.2019121285.
2. Fava, A.; Buyon, J.; Magder, L.; Hodgin, J.; Rosenberg, A.; Demeke, D.S.; Rao, D.A.; Arazi, A.; Celia, A.I.; Putterman, C.; et al. Urine Proteomic Signatures of Histological Class, Activity, Chronicity, and Treatment Response in Lupus Nephritis.
3. Dall'Era, M.; Cisternas, M.G.; Smilek, D.E.; Straub, L.; Houssiau, F.A.; Cervera, R.; Rovin, B.H.; Mackay, M. Predictors of Long-Term Renal Outcome in Lupus Nephritis Trials: Lessons Learned from the Euro-Lupus Nephritis Cohort. *Arthritis Rheumatol.* **2015**, *67*, 1305–1313. https://doi.org/10.1002/art.39026.
4. Tamirou, F.; Lauwerys, B.R.; Dall'Era, M.; Mackay, M.; Rovin, B.; Cervera, R.; Houssiau, F.A. A Proteinuria Cut-off Level of 0.7 g/Day after 12 Months of Treatment Best Predicts Long-Term Renal Outcome in Lupus Nephritis: Data from the *MAINTAIN Nephritis Trial*. *Lupus Sci. Med.* **2015**, *2*, e000123. https://doi.org/10.1136/lupus-2015-000123.
5. Aggarwal, A.; Gupta, R.; Negi, V.S.; Rajasekhar, L.; Misra, R.; Singh, P.; Chaturvedi, V.; Sinha, S. Urinary Haptoglobin, Alpha-1 Anti-Chymotrypsin and Retinol Binding Protein Identified by Proteomics as Potential Biomarkers for Lupus Nephritis. *Clin. Exp. Immunol.* **2017**, *188*, 254–262. https://doi.org/10.1111/cei.12930.
6. Kopetschke, K.; Klocke, J.; Grießbach, A.-S.; Humrich, J.Y.; Biesen, R.; Dragun, D.; Burmester, G.-R.; Enghard, P.; Riemekasten, G. The Cellular Signature of Urinary Immune Cells in Lupus Nephritis: New Insights into Potential Biomarkers. *Arthritis Res. Ther.* **2015**, *17*, 94. https://doi.org/10.1186/s13075-015-0600-y.
7. Jakiela, B.; Kosałka, J.; Plutecka, H.; Węgrzyn, A.S.; Bazan-Socha, S.; Sanak, M.; Musiał, J. Urinary Cytokines and mRNA Expression as Biomarkers of Disease Activity in Lupus Nephritis. *Lupus* **2018**, *27*, 1259–1270. https://doi.org/10.1177/0961203318770006.
8. Alharazy, S.; Kong, N.C.T.; Mohd, M.; Shah, S.A.; Ba'in, A.; Abdul Gafor, A.H. Urine Monocyte Chemoattractant Protein-1 and Lupus Nephritis Disease Activity: Preliminary Report of a Prospective Longitudinal Study. *Autoimmune Dis.* **2015**, *2015*, 1–13. https://doi.org/10.1155/2015/962046.
9. Fatemi, A.; Samadi, G.; Sayedbonakdar, Z.; Smiley, A. Anti-C1q Antibody in Patients with Lupus Nephritic Flare: 18-Month Follow-up and a Nested Case-Control Study. *Mod. Rheumatol.* **2016**, *26*, 233–239. https://doi.org/10.3109/14397595.2015.1074649.
10. Gupta, R.; Yadav, A.; Misra, R.; Aggarwal, A. Urinary sCD25 as a Biomarker of Lupus Nephritis Disease Activity. *Lupus* **2015**, *24*, 273–279. https://doi.org/10.1177/0961203314555174.
11. Kapsia, E.; Marinaki, S.; Michelakis, I.; Liapis, G.; Sfikakis, P.P.; Boletis, J.; Tektonidou, M.G. Predictors of Early Response, Flares, and Long-Term Adverse Renal Outcomes in Proliferative Lupus Nephritis: A 100-Month Median Follow-Up of an Inception Cohort. *J. Clin. Med.* **2022**, *11*, 5017. https://doi.org/10.3390/jcm11175017.
12. Ichinose, K.; Kitamura, M.; Sato, S.; Fujikawa, K.; Horai, Y.; Matsuoka, N.; Tsuboi, M.; Nonaka, F.; Shimizu, T.; Fukui, S.; et al. Podocyte Foot Process Width Is a Prediction Marker for Complete Renal Response at 6 and 12 Months after Induction Therapy in Lupus Nephritis. *Clin. Immunol.* **2018**, *197*, 161–168. https://doi.org/10.1016/j.clim.2018.10.002.
13. Petri, M.; Barr, E.; Magder, L.S. Risk of Renal Failure Within 10 or 20 Years of Systemic Lupus Erythematosus Diagnosis. *J. Rheumatol.* **2021**, *48*, 222–227. https://doi.org/10.3899/jrheum.191094.
14. Calatroni, M.; Conte, E.; Stella, M.; De Liso, F.; Reggiani, F.; Moroni, G. Clinical and Immunological Biomarkers Can Identify Proliferative Changes and Predict Renal Flares in Lupus Nephritis. *Arthritis Res. Ther.* **2025**, *27*, 72. https://doi.org/10.1186/s13075-025-03536-5.

15. Landolt-Marticorena, C.; Prokopec, S.D.; Morrison, S.; Noamani, B.; Bonilla, D.; Reich, H.; Scholey, J.; Avila-Casado, C.; Fortin, P.R.; Boutros, P.C.; et al. A Discrete Cluster of Urinary Biomarkers Discriminates between Active Systemic Lupus Erythematosus Patients with and without Glomerulonephritis. *Arthritis Res. Ther.* **2016**, *18*, 218. https://doi.org/10.1186/s13075-016-1120-0.
16. Yang, Z.; Zhang, Z.; Qin, B.; Wu, P.; Zhong, R.; Zhou, L.; Liang, Y. Human Epididymis Protein 4: A Novel Biomarker for Lupus Nephritis and Chronic Kidney Disease in Systemic Lupus Erythematosus. *J. Clin. Lab. Anal.* **2016**, *30*, 897–904. https://doi.org/10.1002/jcla.21954.
17. Wong, C.C.Y.; Gao, L.Y.; Xu, Y.; Chau, M.K.M.; Zhang, D.; Yap, D.Y.H.; Ying, S.K.Y.; Lee, C.K.; Yung, S.; Chan, T.M. Cluster of Differentiation-44 as a Novel Biomarker of Lupus Nephritis and Its Role in Kidney Inflammation and Fibrosis. *Front. Immunol.* **2024**, *15*, 1443153. https://doi.org/10.3389/fimmu.2024.1443153.
18. Pang, Y.; Tan, Y.; Li, Y.; Zhang, J.; Guo, Y.; Guo, Z.; Zhang, C.; Yu, F.; Zhao, M. Serum A08 C1q Antibodies Are Associated with Disease Activity and Prognosis in Chinese Patients with Lupus Nephritis. *Kidney Int.* **2016**, *90*, 1357–1367. https://doi.org/10.1016/j.kint.2016.08.010.
19. Whittall Garcia, L.P.; Gladman, D.D.; Urowitz, M.; Bonilla, D.; Schneider, R.; Touma, Z.; Wither, J. Interferon-α as a Biomarker to Predict Renal Outcomes in Lupus Nephritis. *Lupus Sci. Med.* **2024**, *11*, e001347. https://doi.org/10.1136/lupus-2024-001347.
20. Chen, Y.M.; Hung, W.T.; Liao, Y.W.; Hsu, C.Y.; Hsieh, T.Y.; Chen, H.H.; Hsieh, C.W.; Lin, C.T.; Lai, K.L.; Tang, K.T.; et al. Combination Immunosuppressant Therapy and Lupus Nephritis Outcome: A Hospital-Based Study. *Lupus* **2019**, *28*, 658–666. https://doi.org/10.1177/0961203319842663.
21. Häyry, A.; Faustini, F.; Zickert, A.; Larsson, A.; Niewold, T.B.; Svenungsson, E.; Oke, V.; Gunnarsson, I. Interleukin (IL) 16: A Candidate Urinary Biomarker for Proliferative Lupus Nephritis. *Lupus Sci. Med.* **2022**, *9*, e000744. https://doi.org/10.1136/lupus-2022-000744.
22. Xu, B.; Zhang, Y.; Yang, Y.; Liu, Y.; Feng, J. Diagnostic Performance of Serum Cystatin C and Complement Component 1q in Lupus Nephritis. *Arthritis Res. Ther.* **2019**, *21*, 267. https://doi.org/10.1186/s13075-019-2065-x.
23. Obrișcă, B.; Vrabie, A.; Lujinschi, Ștefan; Jurubiță, R.; Mocanu, V.; Berechet, A.; Sorohan, B.; Andronesi, A.; Lupușoru, G.; Achim, C.; et al. Clinical Predictors of Underlying Histologic Activity in Patients with Lupus Nephritis: A Focus on Urinary Soluble CD163. *J. Clin. Med.* **2025**, *14*, 6162. https://doi.org/10.3390/jcm14176162.
24. Kitagawa, A.; Tsuboi, N.; Yokoe, Y.; Katsuno, T.; Ikeuchi, H.; Kajiyama, H.; Endo, N.; Sawa, Y.; Suwa, J.; Sugiyama, Y.; et al. Urinary Levels of the Leukocyte Surface Molecule CD11b Associate with Glomerular Inflammation in Lupus Nephritis. *Kidney Int.* **2019**, *95*, 680–692. https://doi.org/10.1016/j.kint.2018.10.025.
25. Mejía-Vilet, J.M.; Córdova-Sánchez, B.M.; Arreola-Guerra, J.M.; Morales-Buenrostro, L.E.; Uribe-Uribe, N.O.; Correa-Rotter, R. Renal Flare Prediction and Prognosis in Lupus Nephritis Hispanic Patients. *Lupus* **2016**, *25*, 315–324. https://doi.org/10.1177/0961203315606985.
26. El-Mohsen, M.A.; Tawfik, A.; Bichari, W.; Shawky, S.; Mady, G.; Hassan, M. Value of Urinary Neutrophil Gelatinase-Associated Lipocalin versus Conventional Biomarkers in Predicting Response to Treatment of Active Lupus Nephritis. *Int. J. Nephrol.* **2020**, *2020*, 1–8. https://doi.org/10.1155/2020/8855614.
27. McDonald, S.; Yiu, S.; Su, L.; Gordon, C.; Truman, M.; Lisk, L.; Solomons, N.; Bruce, I.N. Predictors of Treatment Response in a Lupus Nephritis Population: Lessons from the Aspreva Lupus Management Study (ALMS) Trial. *Lupus Sci. Med.* **2022**, *9*, e000584. https://doi.org/10.1136/lupus-2021-000584.
28. Park, D.J.; Kang, J.H.; Lee, J.W.; Lee, K.E.; Kim, T.J.; Park, Y.W.; Lee, J.S.; Choi, Y.D.; Lee, S.S. Risk Factors to Predict the Development of Chronic Kidney Disease in Patients with Lupus Nephritis. *Lupus* **2017**, *26*, 1139–1148. https://doi.org/10.1177/0961203317694257.
29. Nozaki, Y.; Shiga, T.; Ashida, C.; Tomita, D.; Itami, T.; Kishimoto, K.; Kinoshita, K.; Matsumura, I. U-KIM-1 as a Predictor of Treatment Response in Lupus Nephritis. *Lupus* **2023**, *32*, 54–62. https://doi.org/10.1177/09612033221135871.

30. Koo, H.S.; Kim, S.; Chin, H.J. Remission of Proteinuria Indicates Good Prognosis in Patients with Diffuse Proliferative Lupus Nephritis. *Lupus* **2016**, *25*, 3–11. https://doi.org/10.1177/0961203315595130.
31. Domingues, V.; Levinson, B.A.; Bornkamp, N.; Goldberg, J.D.; Buyon, J.; Belmont, H.M. Serum Albumin at 1 Year Predicts Long-Term Renal Outcome in Lupus Nephritis. *Lupus Sci. Med.* **2018**, *5*, e000271. https://doi.org/10.1136/lupus-2018-000271.
32. Liu, X.-R.; Qi, Y.-Y.; Zhao, Y.-F.; Cui, Y.; Wang, X.-Y.; Zhao, Z.-Z. Albumin-to-Globulin Ratio (AGR) as a Potential Marker of Predicting Lupus Nephritis in Chinese Patients with Systemic Lupus Erythematosus. *Lupus* **2021**, *30*, 412–420. https://doi.org/10.1177/0961203320981139.
33. Kwon, O.C.; Lee, E.-J.; Oh, J.S.; Hong, S.; Lee, C.-K.; Yoo, B.; Park, M.-C.; Kim, Y.-G. Plasma Immunoglobulin Binding Protein 1 as a Predictor of Development of Lupus Nephritis. *Lupus* **2020**, *29*, 547–553. https://doi.org/10.1177/0961203320912336.
34. Mok, C.C.; Ding, H.H.; Kharboutli, M.; Mohan, C. Axl, Ferritin, Insulin-Like Growth Factor Binding Protein 2, and Tumor Necrosis Factor Receptor Type II as Biomarkers in Systemic Lupus Erythematosus. *Arthritis Care Res.* **2016**, *68*, 1303–1309. https://doi.org/10.1002/acr.22835.
35. Hardt, U.; Larsson, A.; Gunnarsson, I.; Clancy, R.M.; Petri, M.; Buyon, J.P.; Silverman, G.J.; Svenungsson, E.; Grönwall, C. Autoimmune Reactivity to Malondialdehyde Adducts in Systemic Lupus Erythematosus Is Associated with Disease Activity and Nephritis. *Arthritis Res. Ther.* **2018**, *20*, 36. https://doi.org/10.1186/s13075-018-1530-2.
36. Barnado, A.; Carroll, R.J.; Casey, C.; Wheless, L.; Denny, J.C.; Crofford, L.J. Phenome-Wide Association Study Identifies dsDNA as a Driver of Major Organ Involvement in Systemic Lupus Erythematosus. *Lupus* **2019**, *28*, 66–76. https://doi.org/10.1177/0961203318815577.
37. Plawecki, M.; Lheritier, E.; Clavarino, G.; Jourde-Chiche, N.; Ouili, S.; Paul, S.; Gout, E.; Sarrot-Reynauld, F.; Bardin, N.; Boëlle, P.-Y.; et al. Association between the Presence of Autoantibodies Targeting Ficolin-3 and Active Nephritis in Patients with Systemic Lupus Erythematosus. *PLOS ONE* **2016**, *11*, e0160879. https://doi.org/10.1371/journal.pone.0160879.
38. Gargiulo, M.D.L.Á.; Khoury, M.; Gómez, G.; Grimaudo, S.; Suárez, L.; Collado, M.V.; Sarano, J. Cut-off Values of Immunological Tests to Identify Patients at High Risk of Severe Lupus Nephritis. *Medicina (Mex.)* **2018**, *78*, 329–335.
39. Sjöwall, C.; Bentow, C.; Aure, M.A.; Mahler, M. Two-Parametric Immunological Score Development for Assessing Renal Involvement and Disease Activity in Systemic Lupus Erythematosus. *J. Immunol. Res.* **2018**, *2018*, 1–9. https://doi.org/10.1155/2018/1294680.
40. Kwon, O.C.; Lee, J.S.; Ghang, B.; Kim, Y.-G.; Lee, C.-K.; Yoo, B.; Hong, S. Predicting Eventual Development of Lupus Nephritis at the Time of Diagnosis of Systemic Lupus Erythematosus. *Semin. Arthritis Rheum.* **2018**, *48*, 462–466. https://doi.org/10.1016/j.semarthrit.2018.02.012.
41. Bruschi, M.; Moroni, G.; Sinico, R.A.; Franceschini, F.; Fredi, M.; Vaglio, A.; Cavagna, L.; Petretto, A.; Pratesi, F.; Migliorini, P.; et al. Serum IgG2 Antibody Multicomposition in Systemic Lupus Erythematosus and Lupus Nephritis (Part 1): Cross-Sectional Analysis. *Rheumatology* **2021**, *60*, 3176–3188. https://doi.org/10.1093/rheumatology/keaa767.
42. Gupta, R.; Yadav, A.; Aggarwal, A. Urinary Soluble CD163 Is a Good Biomarker for Renal Disease Activity in Lupus Nephritis. *Clin. Rheumatol.* **2021**, *40*, 941–948. https://doi.org/10.1007/s10067-020-05343-6.
43. Gupta, R.; Yadav, A.; Aggarwal, A. Longitudinal Assessment of Monocyte Chemoattractant Protein-1 in Lupus Nephritis as a Biomarker of Disease Activity. *Clin. Rheumatol.* **2016**, *35*, 2707–2714. https://doi.org/10.1007/s10067-016-3404-9.
44. Mok, C.C.; Solimar, S.; Ho, L.Y.; Mohamed, F.A.; Mohamed, F.I.; Mohan, C. Urinary Angiostatin, CXCL4 and VCAM-1 as Biomarkers of Lupus Nephritis. *Arthritis Res. Ther.* **2018**, *20*. https://doi.org/10.1186/s13075-017-1498-3.

45. Renaudineau, Y.; Chauveau, D.; Faguer, S.; Huart, A.; Ribes, D.; Pugnet, G.; Sailler, L.; Jamme, T.; Treiner, E.; Fortenfant, F.; et al. Urinary Soluble CD163 Is Useful as “Liquid Biopsy” Marker in Lupus Nephritis at Both Diagnosis and Follow-up to Predict Impending Flares. *J. Transl. Autoimmun.* **2024**, *9*, 100244. https://doi.org/10.1016/j.jtauto.2024.100244.
46. Fava, A.; Wagner, C.A.; Guthridge, C.J.; Kheir, J.; Macwana, S.; DeJager, W.; Gross, T.; Izmirly, P.; Belmont, H.M.; Diamond, B.; et al. Association of Autoantibody Concentrations and Trajectories With Lupus Nephritis Histologic Features and Treatment Response. *Arthritis Rheumatol.* **2024**, *76*, 1611–1622. https://doi.org/10.1002/art.42941.
47. Vasilev, V.; Artero, M.R.; Petkova, M.; Mihaylova, G.; Dragon-Durey, M.-A.; Radanova, M.; Roumenina, L.T. Clinical Relevance of Anti-C3 and Anti-C4 Autoantibodies in Lupus Nephritis. *Kidney Int. Rep.* **2024**, *9*, 1429–1440. https://doi.org/10.1016/j.ekir.2024.01.052.
48. Kianmehr, N.; Khoshmirsafa, M.; Shekarabi, M.; Falak, R.; Haghighi, A.; Masoodian, M.; Seif, F.; Omidi, F.; Shirani, F.; Dadfar, N. High Frequency of Concurrent Anti-C1q and Anti-dsDNA but Not Anti-C3b Antibodies in Patients with Lupus Nephritis. *J. Immunoassay Immunochem.* **2021**, *42*, 406–423. https://doi.org/10.1080/15321819.2021.1895215.
49. Moroni, G.; Quaglini, S.; Radice, A.; Trezzi, B.; Raffiotta, F.; Messa, P.; Sinico, R.A. The Value of a Panel of Autoantibodies for Predicting the Activity of Lupus Nephritis at Time of Renal Biopsy. *J. Immunol. Res.* **2015**, *2015*, 1–8. https://doi.org/10.1155/2015/106904.
50. Zhang, T.; Li, H.; Vanarsa, K.; Gidley, G.; Mok, C.C.; Petri, M.; Saxena, R.; Mohan, C. Association of Urine sCD163 With Proliferative Lupus Nephritis, Fibrinoid Necrosis, Cellular Crescents and Intrarenal M2 Macrophages. *Front. Immunol.* **2020**, *11*, 671. https://doi.org/10.3389/fimmu.2020.00671.
51. Li, Z.; Sun, Y.; Wang, Y.; Liu, F.; Pan, S.; Li, S.; Guo, Z.; Gao, D.; Yang, J.; Liu, Z.; et al. Proteomics Uncovers ICAM2 (CD102) as a Novel Serum Biomarker of Proliferative Lupus Nephritis. *Lupus Sci. Med.* **2025**, *12*, e001446. https://doi.org/10.1136/lupus-2024-001446.
52. Radanova, M.; Vasilev, V.; Mihaylova, G.; Kosturkova, M.; Kishore, U.; Roumenina, L. Autoantibodies against Complement Classical Pathway Components C1q, C1r, C1s and C1-Inh in Patients with Lupus Nephritis. *Int. J. Mol. Sci.* **2022**, *23*, 9281. https://doi.org/10.3390/ijms23169281.
53. Fasano, S.; Pierro, L.; Borgia, A.; Coscia, M.A.; Formica, R.; Bucci, L.; Riccardi, A.; Ciccia, F. Biomarker Panels May Be Superior over Single Molecules in Prediction of Renal Flares in Systemic Lupus Erythematosus: An Exploratory Study. *Rheumatology* **2020**, *59*, 3193–3200. https://doi.org/10.1093/rheumatology/keaa074.
54. Himbert, M.; Jourde-Chiche, N.; Chapart, L.; Charles, N.; Baumstarck, K.; Daugas, E. Anti-dsDNA IgE: A Potential Non-Invasive Test for Prediction of Lupus Nephritis Relapse. *RMD Open* **2024**, *10*, e004255. https://doi.org/10.1136/rmdopen-2024-004255.
55. Pérez-Isidro, A.; Xipell, M.; Llobell, A.; De Moner, N.; Lledó, G.M.; Cervera, R.; Prieto-González, S.; Quintana, L.F.; Espinosa, G.; García-Ormaechea, M.; et al. Anti-dsDNA B-Cell ELISpot as a Monitoring and Flare Prediction Tool in SLE Patients. *J. Clin. Med.* **2023**, *12*, 1295. https://doi.org/10.3390/jcm12041295.
56. Kim, J.; Lee, J.S.; Go, H.; Lim, J.S.; Oh, J.S.; Kim, Y.-G.; Lee, C.-K.; Yoo, B.; Hong, S. Clinical and Histological Significance of Urinary CD11c+ Macrophages in Lupus Nephritis. *Arthritis Res. Ther.* **2020**, *22*, 173. https://doi.org/10.1186/s13075-020-02265-1.
57. Colliard, S.; Jourde-Chiche, N.; Clavarino, G.; Sarrot-Reynauld, F.; Gout, E.; Deroux, A.; Fougere, M.; Bardin, N.; Bouillet, L.; Cesbron, J.; et al. Autoantibodies Targeting Ficolin-2 in Systemic Lupus Erythematosus Patients With Active Nephritis. *Arthritis Care Res.* **2018**, *70*, 1263–1268. https://doi.org/10.1002/acr.23449.
58. Birmingham, D.J.; Bitter, J.E.; Ndukwe, E.G.; Dials, S.; Gullo, T.R.; Conroy, S.; Nagaraja, H.N.; Rovin, B.H.; Hebert, L.A. Relationship of Circulating Anti-C3b and Anti-C1q IgG to Lupus Nephritis and Its Flare. *Clin. J. Am. Soc. Nephrol.* **2016**, *11*, 47–53. https://doi.org/10.2215/CJN.03990415.

59. Chi, S.; Yu, Y.; Shi, J.; Zhang, Y.; Yang, J.; Yang, L.; Liu, X. Antibodies against C1q Are a Valuable Serological Marker for Identification of Systemic Lupus Erythematosus Patients with Active Lupus Nephritis. *Dis. Markers* **2015**, *2015*, 1–11. https://doi.org/10.1155/2015/450351.
60. Gómez-Puerta, J.A.; Ortiz-Reyes, B.; Urrego, T.; Vanegas-García, A.L.; Muñoz, C.H.; González, L.A.; Cervera, R.; Vásquez, G. Urinary Neutrophil Gelatinase-Associated Lipocalin and Monocyte Chemoattractant Protein 1 as Biomarkers for Lupus Nephritis in Colombian SLE Patients. *Lupus* **2018**, *27*, 637–646. https://doi.org/10.1177/0961203317738226.
61. Jia, Y.; Zhao, L.; Wang, C.; Shang, J.; Miao, Y.; Dong, Y.; Zhao, Z. Anti-Double-Stranded DNA Isotypes and Anti-C1q Antibody Improve the Diagnostic Specificity of Systemic Lupus Erythematosus. *Dis. Markers* **2018**, *2018*, 1–7. https://doi.org/10.1155/2018/4528547.
62. Ishizaki, J.; Saito, K.; Nawata, M.; Mizuno, Y.; Tokunaga, M.; Sawamukai, N.; Tamura, M.; Hirata, S.; Yamaoka, K.; Hasegawa, H.; et al. Low Complements and High Titre of Anti-Sm Antibody as Predictors of Histopathologically Proven Silent Lupus Nephritis without Abnormal Urinalysis in Patients with Systemic Lupus Erythematosus. *Rheumatology* **2015**, *54*, 405–412. https://doi.org/10.1093/rheumatology/keu343.
63. Bock, M.; Heijnen, I.; Trendelenburg, M. Anti-C1q Antibodies as a Follow-Up Marker in SLE Patients. *PLOS ONE* **2015**, *10*, e0123572. https://doi.org/10.1371/journal.pone.0123572.
64. Vigne, J.; Haut, N.; Clavarino, G.; Jourde-Chiche, N.; Sarrot-Reynauld, F.; Trouw, L.A.; Defendi, F.; Thielens, N.M.; Gaboriaud, C.; Rossi, V.; et al. Anti-C1s Autoantibodies as Complementary Serologic Biomarker in Lupus Nephritis. *Clin. Immunol.* **2025**, *275*, 110487. https://doi.org/10.1016/j.clim.2025.110487.
65. Zhao, L.; Wang, W.; Wu, L.; Wu, T.; Tu, J.; Wu, X.; Sun, F.; Ding, H.; Shen, N.; Wu, H.; et al. Combination of Anti-SSA/Ro60 and Anti-dsDNA Serotype Is Predictive of Belimumab Renal Response in Patients with Lupus Nephritis. *Lupus Sci. Med.* **2024**, *11*, e001156. https://doi.org/10.1136/lupus-2024-001156.
66. Martin, M.; Trattner, R.; Nilsson, S.C.; Björk, A.; Zickert, A.; Blom, A.M.; Gunnarsson, I. Plasma C4d Correlates With C4d Deposition in Kidneys and With Treatment Response in Lupus Nephritis Patients. *Front. Immunol.* **2020**, *11*, 582737. https://doi.org/10.3389/fimmu.2020.582737.
67. Phatak, S.; Chaurasia, S.; Mishra, S.K.; Gupta, R.; Agrawal, V.; Aggarwal, A.; Misra, R. Urinary B Cell Activating Factor (BAFF) and a Proliferation-Inducing Ligand (APRIL): Potential Biomarkers of Active Lupus Nephritis. *Clin. Exp. Immunol.* **2017**, *187*, 376–382. https://doi.org/10.1111/cei.12894.
68. Ganguly, S.; Majumder, S.; Kumar, S.; Gupta, R.; Muhammed, H.; Shobha, V.; Aggarwal, A.; Misra, R. Urinary C3d Is Elevated in Patients with Active Lupus Nephritis and a Fall in Its Level after 3 Months Predicts Response at 6 Months on Follow Up. *Lupus* **2020**, *29*, 1800–1806. https://doi.org/10.1177/0961203320950019.
69. Selvaraja, M.; Abdullah, M.; Arip, M.; Chin, V.K.; Shah, A.; Amin Nordin, S. Elevated Interleukin-25 and Its Association to Th2 Cytokines in Systemic Lupus Erythematosus with Lupus Nephritis. *PLOS ONE* **2019**, *14*, e0224707. https://doi.org/10.1371/journal.pone.0224707.
70. Ding, H.; Lin, C.; Cai, J.; Guo, Q.; Dai, M.; Mohan, C.; Shen, N. Urinary Activated Leukocyte Cell Adhesion Molecule as a Novel Biomarker of Lupus Nephritis Histology. *Arthritis Res. Ther.* **2020**, *22*, 122. https://doi.org/10.1186/s13075-020-02209-9.
71. Liang, P.; Tang, Y.; Lin, L.; Zhong, H.; Yang, H.; Zeng, Y.; Lv, J.; Li, X.; Lu, Y.; Xu, A. Low Level of Circulating Basophil Counts in Biopsy-Proven Active Lupus Nephritis. *Clin. Rheumatol.* **2018**, *37*, 459–465. https://doi.org/10.1007/s10067-017-3858-4.
72. Ruchakorn, N.; Ngamjanyaporn, P.; Suangtamai, T.; Kafaksom, T.; Polpanumas, C.; Petpisit, V.; Pisitkun, T.; Pisitkun, P. Performance of Cytokine Models in Predicting SLE Activity. *Arthritis Res. Ther.* **2019**, *21*, 287. https://doi.org/10.1186/s13075-019-2029-1.
73. Buyon, J.P.; Kim, M.Y.; Guerra, M.M.; Lu, S.; Reeves, E.; Petri, M.; Laskin, C.A.; Lockshin, M.D.; Sammaritano, L.R.; Branch, D.W.; et al. Kidney Outcomes and Risk Factors for Nephritis (Flare/De Novo) in a Multiethnic Cohort of Pregnant Patients with Lupus. *Clin. J. Am. Soc. Nephrol.* **2017**, *12*, 940–946. https://doi.org/10.2215/CJN.11431116.

74. Rossi, G.M. Persistent Isolated C3 Hypocomplementemia as a Strong Predictor of End-Stage Kidney Disease in Lupus Nephritis.
75. Parodis, I.; Zickert, A.; Sundelin, B.; Axelsson, M.; Gerhardsson, J.; Svenungsson, E.; Malmström, V.; Gunnarsson, I. Evaluation of B Lymphocyte Stimulator and a Proliferation Inducing Ligand as Candidate Biomarkers in Lupus Nephritis Based on Clinical and Histopathological Outcome Following Induction Therapy. *Lupus Sci. Med.* **2015**, *2*, e000061. https://doi.org/10.1136/lupus-2014-000061.
76. Li, Y.; Tang, C.; Vanarsa, K.; Thai, N.; Castillo, J.; Lea, G.A.B.; Lee, K.H.; Kim, S.; Pedroza, C.; Wu, T.; et al. Proximity Extension Assay Proteomics and Renal Single Cell Transcriptomics Uncover Novel Urinary Biomarkers for Active Lupus Nephritis. *J. Autoimmun.* **2024**, *143*, 103165. https://doi.org/10.1016/j.jaut.2023.103165.
77. Vincent, F.B.; Kandane-Rathnayake, R.; Hoi, A.Y.; Slavin, L.; Godsell, J.D.; Kitching, A.R.; Harris, J.; Nelson, C.L.; Jenkins, A.J.; Chrysostomou, A.; et al. Urinary B-Cell-Activating Factor of the Tumour Necrosis Factor Family (BAFF) in Systemic Lupus Erythematosus. *Lupus* **2018**, *27*, 2029–2040. https://doi.org/10.1177/0961203318804885.
78. Abdel Galil, S.M.; Ezzeldin, N.; El-Boshy, M.E. The Role of Serum IL-17 and IL-6 as Biomarkers of Disease Activity and Predictors of Remission in Patients with Lupus Nephritis. *Cytokine* **2015**, *76*, 280–287. https://doi.org/10.1016/j.cyto.2015.05.007.
79. Dedong, H.; Feiyan, Z.; Jie, S.; Xiaowei, L.; Shaoyang, W. Analysis of Interleukin-17 and Interleukin-23 for Estimating Disease Activity and Predicting the Response to Treatment in Active Lupus Nephritis Patients. *Immunol. Lett.* **2019**, *210*, 33–39. https://doi.org/10.1016/j.imlet.2019.04.002.
80. Nordin, F.; Shaharir, S.S.; Abdul Wahab, A.; Mustafar, R.; Abdul Gafor, A.H.; Mohamed Said, M.S.; Rajalingham, S.; Shah, S.A. Serum and Urine interleukin-17A Levels as Biomarkers of Disease Activity in Systemic Lupus Erythematosus. *Int. J. Rheum. Dis.* **2019**, *22*, 1419–1426. https://doi.org/10.1111/1756-185X.13615.
81. Saif, D.S.; Abdelsattar, S.; Zahran, E.S.; Khalil, M.; Samir, S.; Abo Mansour, H.E. Interleukin Biomarkers as Predictive Tools for Lupus Nephritis Grade and Disease Activity in Systemic Lupus Erythematosus. *ARP Rheumatol.* **2025**, *4*, 80–90.
82. Salem, M.N.; Taha, H.A.; Abd El-Fattah El-Feqi, M.; Eesa, N.N.; Mohamed, R.A. Urinary TNF-like Weak Inducer of Apoptosis (TWEAK) as a Biomarker of Lupus Nephritis. *Z. Für Rheumatol.* **2018**, *77*, 71–77. https://doi.org/10.1007/s00393-016-0184-1.
83. Susianti, H.; Hanggara, D.S.; Lestari, K.D.; Purnamasari, P.; Aprilia, A. Analysis of TNF-like Weak Inducer of Apoptosis for Detecting Lupus Nephritis. *Comp. Clin. Pathol.* **2022**, *31*, 313–316. https://doi.org/10.1007/s00580-022-03334-4.
84. Dong, X.W.; Zheng, Z.H.; Ding, J.; Luo, X.; Li, Z.Q.; Li, Y.; Rong, M.Y.; Fu, Y.L.; Shi, J.H.; Yu, L.C.; et al. Combined Detection of uMCP-1 and uTWEAK for Rapid Discrimination of Severe Lupus Nephritis. *Lupus* **2018**, *27*, 971–981. https://doi.org/10.1177/0961203318758507.
85. Reyes-Martínez, F.; Pérez-Navarro, M.; Rodríguez-Matías, A.; Soto-Abraham, V.; Gutierrez-Reyes, G.; Medina-Avila, Z.; Valdez-Ortiz, R. Assessment of Urinary TWEAK Levels in Mexican Patients with Untreated Lupus Nephritis: An Exploratory Study. *Nefrología* **2018**, *38*, 152–160. https://doi.org/10.1016/j.nefro.2017.04.005.
86. Elsaid, D.S.; Abdel Noor, R.A.; Shalaby, K.A.; Haroun, R.A.-H. Urinary Tumor Necrosis Factor-Like Weak Inducer of Apoptosis (uTWEAK) and Urinary Monocyte Chemo-Attractant Protein-1 (uMCP-1): Promising Biomarkers of Lupus Nephritis Activity? *Saudi J. Kidney Dis. Transplant. Off. Publ. Saudi Cent. Organ Transplant. Saudi Arab.* **2021**, *32*, 19–29. https://doi.org/10.4103/1319-2442.318522.
87. Selim, Z.I.; Khader, T.M.; Mohammed, H.O.; Al-Hammady, D.H.; Seif, H.M.A.; Al-Johi, A.A. Urinary Tumor Necrosis Factor-Like Weak Inducer of Apoptosis (uTWEAK) as a Biomarker for Lupus Nephritis Activity and Its Correlation with Histolopathological Findings of Renal Biopsy. *Egypt. Rheumatol.* **2019**, *41*, 19–23. https://doi.org/10.1016/j.ejr.2018.01.008.
88. Abediazar, S.; Jafari-Nakhjavani, M.; Ghorbanihaghjo, A.; Shekarchi, M.; Zununi-Vahed, S. Serum Levels of CXCL10 and Vitamin D in Patients with Lupus Nephritis. **2019**, *13*.

89. Klocke, J.; Kopetschke, K.; Grießbach, A.; Langhans, V.; Humrich, J.Y.; Biesen, R.; Dragun, D.; Radbruch, A.; Burmester, G.; Riemekasten, G.; et al. Mapping Urinary Chemokines in Human Lupus Nephritis: Potentially Redundant Pathways Recruit CD4+ and CD8+ T Cells and Macrophages. *Eur. J. Immunol.* **2017**, *47*, 180–192. https://doi.org/10.1002/eji.201646387.
90. Stanley, S.; Mok, C.C.; Vanarsa, K.; Habazi, D.; Li, J.; Pedroza, C.; Saxena, R.; Mohan, C. Identification of Low-Abundance Urinary Biomarkers in Lupus Nephritis Using Electrochemiluminescence Immunoassays. *Arthritis Rheumatol.* **2019**, *71*, 744–755. https://doi.org/10.1002/art.40813.
91. Wang, S.; Cui, Y. Clinical Significance of Serum CXCL9, CXCL10, and CXCL11 in Patients with Lupus Nephritis. *Immun. Inflamm. Dis.* **2024**, *12*, e1368. https://doi.org/10.1002/iid3.1368.
92. Dong, X.; Zheng, Z.; Luo, X.; Ding, J.; Li, Y.; Li, Z.; Li, S.; Rong, M.; Fu, Y.; Wu, Z.; et al. Combined Utilization of Untimed Single Urine of MCP-1 and TWEAK as a Potential Indicator for Proteinuria in Lupus Nephritis: A Case–Control Study. *Medicine (Baltimore)* **2018**, *97*, e0343. https://doi.org/10.1097/MD.0000000000010343.
93. Stanley, S.; Vanarsa, K.; Soliman, S.; Habazi, D.; Pedroza, C.; Gidley, G.; Zhang, T.; Mohan, S.; Der, E.; Suryawanshi, H.; et al. Comprehensive Aptamer-Based Screening Identifies a Spectrum of Urinary Biomarkers of Lupus Nephritis across Ethnicities. *Nat. Commun.* **2020**, *11*, 2197. https://doi.org/10.1038/s41467-020-15986-3.
94. Whittall-Garcia, L.; Goliad, K.; Kim, M.; Bonilla, D.; Gladman, D.; Urowitz, M.; Fortin, P.R.; Atenafu, E.G.; Touma, Z.; Wither, J. Identification and Validation of a Urinary Biomarker Panel to Accurately Diagnose and Predict Response to Therapy in Lupus Nephritis. *Front. Immunol.* **2022**, *13*, 889931. https://doi.org/10.3389/fimmu.2022.889931.
95. Liu, L.; Wang, R.; Ding, H.; Tian, L.; Gao, T.; Bao, C. The Utility of Urinary Biomarker Panel in Predicting Renal Pathology and Treatment Response in Chinese Lupus Nephritis Patients. *PLOS ONE* **2020**, *15*, e0240942. https://doi.org/10.1371/journal.pone.0240942.
96. Endo, N.; Tsuboi, N.; Furuhashi, K.; Shi, Y.; Du, Q.; Abe, T.; Hori, M.; Imaizumi, T.; Kim, H.; Katsuno, T.; et al. Urinary Soluble CD163 Level Reflects Glomerular Inflammation in Human Lupus Nephritis. *Nephrol. Dial. Transplant.* **2016**, *31*, 2023–2033. https://doi.org/10.1093/ndt/gfw214.
97. Bona, N.; Pezzarini, E.; Balbi, B.; Daniele, S.M.; Rossi, M.F.; Monje, A.L.; Basiglio, C.; Pelusa, H.F.; Arriaga, S.M.M. Oxidative Stress, Inflammation and Disease Activity Biomarkers in Lupus Nephropathy. *Lupus* **2020**, *29*, 311–323. https://doi.org/10.1177/0961203320904784.
98. Ngamjanyaporn, P.; Worawichawong, S.; Pisitkun, P.; Khiewngam, K.; Kantachuvesiri, S.; Nongnuch, A.; Assanatham, M.; Sathirapongsasuti, N.; Kitiyakara, C. Predicting Treatment Response and Clinicopathological Findings in Lupus Nephritis with Urine Epidermal Growth Factor, Monocyte Chemoattractant Protein-1 or Their Ratios. *PLOS ONE* **2022**, *17*, e0263778. https://doi.org/10.1371/journal.pone.0263778.
99. Lai, L.; Wu, C.; Li, X.; Rong, Y.; Huang, Y.; Wang, B. Urinary MCP-1 and VCAM-1 as Non-Invasive Biomarkers for the Diagnosis and Activity Assessment of Lupus Nephritis. *PLOS One* **2025**, *20*, e0323334. https://doi.org/10.1371/journal.pone.0323334.
100. Davies, J.C.; Carlsson, E.; Midgley, A.; Smith, E.M.D.; Bruce, I.N.; Beresford, M.W.; Hedrich, C.M.; the BILAG-BR and MRC MASTERPLANS Consortia A Panel of Urinary Proteins Predicts Active Lupus Nephritis and Response to Rituximab Treatment. *Rheumatology* **2021**, *60*, 3747–3759. https://doi.org/10.1093/rheumatology/keaa851.
101. Soliman, S.A.; Stanley, S.; Vanarsa, K.; Ismail, F.; Mok, C.C.; Mohan, C. Exploring Urine:Serum Fractional Excretion Ratios as Potential Biomarkers for Lupus Nephritis. *Front. Immunol.* **2022**, *13*, 910993. https://doi.org/10.3389/fimmu.2022.910993.
102. Chalmers, S.A.; Ayilam Ramachandran, R.; Garcia, S.J.; Der, E.; Herlitz, L.; Ampudia, J.; Chu, D.; Jordan, N.; Zhang, T.; Parodis, I.; et al. The CD6/ALCAM Pathway Promotes Lupus Nephritis via T Cell–Mediated Responses. *J. Clin. Invest.* **2022**, *132*, e147334. https://doi.org/10.1172/JCI147334.

103. Amer, A.S.; Abdel Moneam, S.M.; Hashaad, N.I.; Yousef, E.M.; Abd El-Hassib, D.M. Clinico-Serological Associations of Urinary Activated Leukocyte Cell Adhesion Molecule in Systemic Lupus Erythematosus and Lupus Nephritis. *Clin. Rheumatol.* **2024**, *43*, 1015–1021. https://doi.org/10.1007/s10067-024-06883-x.
104. Kim, J.-W.; Baek, W.-Y.; Jung, J.-Y.; Kim, H.-A.; Lee, S.-W.; Suh, C.-H. Longitudinal Assessment of Urinary ALCAM, HPX, and PRDX6 in Korean Patients with Systemic Lupus Erythematosus: Implications for Disease Activity Monitoring and Treatment Response. *Front. Immunol.* **2024**, *15*, 1369385. https://doi.org/10.3389/fimmu.2024.1369385.
105. Yu, K.Y.; Yung, S.; Chau, M.K.; Tang, C.S.; Yap, D.Y.; Tang, A.H.; Ying, S.K.; Lee, C.K.; Chan, T.M. Clinico-Pathological Associations of Serum VCAM-1 and ICAM-1 Levels in Patients with Lupus Nephritis. *Lupus* **2021**, *30*, 1039–1050. https://doi.org/10.1177/09612033211004727.
106. Gasparin, A.A.; De Andrade, N.P.B.; Hax, V.; Palominos, P.E.; Siebert, M.; Marx, R.; Schaefer, P.G.; Veronese, F.V.; Monticielo, O.A. Urinary Soluble VCAM-1 Is a Useful Biomarker of Disease Activity and Treatment Response in Lupus Nephritis. *BMC Rheumatol.* **2020**, *4*, 67. https://doi.org/10.1186/s41927-020-00162-3.
107. Parodis, I.; Gokaraju, S.; Zickert, A.; Vanarsa, K.; Zhang, T.; Habazi, D.; Botto, J.; Serdoura Alves, C.; Giannopoulos, P.; Larsson, A.; et al. ALCAM and VCAM-1 as Urine Biomarkers of Activity and Long-Term Renal Outcome in Systemic Lupus Erythematosus. *Rheumatology* **2020**, *59*, 2237–2249. https://doi.org/10.1093/rheumatology/kez528.
108. Gamal, N.M.; Badawy, E.R.; Talaat, E.A.; Ibrahim, H.M.; Abd Elsamea, M.H. Clinical Utility of Urinary Soluble CD163 in Evaluation of Lupus Nephritis Patients. *Egypt. Rheumatol.* **2022**, *44*, 151–157. https://doi.org/10.1016/j.ejr.2021.10.003.
109. Yang, G.; Guo, N.; Yin, J.; Wu, J. Elevated Soluble CD163 Predicts Renal Function Deterioration in Lupus Nephritis: A Cohort Study in Eastern China. *J. Int. Med. Res.* **2021**, *49*, 03000605211049963. https://doi.org/10.1177/03000605211049963.
110. Fava, A.; Rao, D.A.; Mohan, C.; Zhang, T.; Rosenberg, A.; Fenaroli, P.; Belmont, H.M.; Izmirly, P.; Clancy, R.; Trujillo, J.M.; et al. Urine Proteomics and Renal SINGLE-CELL Transcriptomics Implicate Interleukin-16 in Lupus Nephritis. *Arthritis Rheumatol.* **2022**, *74*, 829–839. https://doi.org/10.1002/art.42023.
111. Susianti, H.; Iriane, V.M.; Dharmanata, S.; Handono, K.; Widijanti, A.; Gunawan, A.; Kalim, H. Analysis of Urinary TGF-B1, MCP-1, NGAL, and IL-17 as Biomarkers for Lupus Nephritis. *Pathophysiology* **2015**, *22*, 65–71. https://doi.org/10.1016/j.pathophys.2014.12.003.
112. Li, Y.-J.; Wu, H.-H.; Liu, S.-H.; Tu, K.-H.; Lee, C.-C.; Hsu, H.-H.; Chang, M.-Y.; Yu, K.-H.; Chen, W.; Tian, Y.-C. Polyomavirus BK, BKV microRNA, and Urinary Neutrophil Gelatinase-Associated Lipocalin Can Be Used as Potential Biomarkers of Lupus Nephritis. *PLOS ONE* **2019**, *14*, e0210633. https://doi.org/10.1371/journal.pone.0210633.
113. Ibrahim, W.H.M.; Sabry, A.A.; Abdelmoneim, A.R.; Marzouk, H.F.A.; AbdelFattah, R.M. Urinary Neutrophil Gelatinase-Associated Lipocalin (uNGAL) and Kidney Injury Molecule-1 (uKIM-1) as Markers of Active Lupus Nephritis. *Clin. Rheumatol.* **2024**, *43*, 167–174. https://doi.org/10.1007/s10067-023-06698-2.
114. El Shahawy, M.S.; Hemida, M.H.; Abdel-Hafez, H.A.; El-Baz, T.Z.; Lotfy, A.-W.M.; Emran, T.M. Urinary Neutrophil Gelatinase-Associated Lipocalin as a Marker for Disease Activity in Lupus Nephritis. *Scand. J. Clin. Lab. Invest.* **2018**, *78*, 264–268. https://doi.org/10.1080/00365513.2018.1449242.
115. Susianti, H.; Wijaya, J.W.; Rastini, A.; Handono, K.; Gunawan, A.; Kalim, H. Urinary Neutrophil Gelatinase-Associated Lipocalin to Monitor Lupus Nephritis Disease Activity. *Biomark. Insights* **2015**, *10*, BMI.S27625. https://doi.org/10.4137/BMI.S27625.
116. Elewa, E.A.; El Tokhy, M.A.; Fathy, S.E.; Talaat, A.M. Predictive Role of Urinary Neutrophil Gelatinase-Associated Lipocalin in Lupus Nephritis. *Lupus* **2015**, *24*, 138–146. https://doi.org/10.1177/0961203314550225.

117. Satirapoj, B.; Kitiyakara, C.; Leelahavanichkul, A.; Avihingsanon, Y.; Supasyndh, O. Urine Neutrophil Gelatinase-Associated Lipocalin to Predict Renal Response after Induction Therapy in Active Lupus Nephritis. *BMC Nephrol.* **2017**, *18*, 263. https://doi.org/10.1186/s12882-017-0678-3.
118. the Accelerating Medicines Partnership in SLE network; Arazi, A.; Rao, D.A.; Berthier, C.C.; Davidson, A.; Liu, Y.; Hoover, P.J.; Chicoine, A.; Eisenhaure, T.M.; Jonsson, A.H.; et al. The Immune Cell Landscape in Kidneys of Patients with Lupus Nephritis. *Nat. Immunol.* **2019**, *20*, 902–914. https://doi.org/10.1038/s41590-019-0398-x.
119. Lu, J.; Hu, Z.B.; Chen, P.P.; Lu, C.C.; Zhang, J.X.; Li, X.Q.; Yuan, B.Y.; Huang, S.J.; Ma, K.L. Urinary Podocyte Microparticles Are Associated with Disease Activity and Renal Injury in Systemic Lupus Erythematosus. *BMC Nephrol.* **2019**, *20*, 303. https://doi.org/10.1186/s12882-019-1482-z.
120. Burbano, C.; Gómez-Puerta, J.A.; Muñoz-Vahos, C.; Vanegas-García, A.; Rojas, M.; Vásquez, G.; Castaño, D. HMGB1^{+} Microparticles Present in Urine Are Hallmarks of Nephritis in Patients with Systemic Lupus Erythematosus. *Eur. J. Immunol.* **2019**, *49*, 323–335. https://doi.org/10.1002/eji.201847747.
121. ElFeky, D.S.; Omar, N.M.; Shaker, O.G.; Abdelrahman, W.; Gheita, T.A.; Nada, M.G. Circulatory microRNAs and Proinflammatory Cytokines as Predictors of Lupus Nephritis. *Front. Immunol.* **2024**, *15*, 1449296. https://doi.org/10.3389/fimmu.2024.1449296.
122. Khoshmirsafa, M.; Kianmehr, N.; Falak, R.; Mowla, S.J.; Seif, F.; Mirzaei, B.; Valizadeh, M.; Shekarabi, M. Elevated Expression of miR-21 and miR-155 in Peripheral Blood Mononuclear Cells as Potential Biomarkers for Lupus Nephritis. *Int. J. Rheum. Dis.* **2019**, *22*, 458–467. https://doi.org/10.1111/1756-185X.13410.
123. Nakhjavani, M.; Etemadi, J.; Pourlak, T.; Mirhosaini, Z.; Zununi Vahed, S.; Abediazar, S. Plasma Levels of miR-21, miR-150, miR-423 in Patients with Lupus Nephritis. *Iran. J. Kidney Dis.* **2019**, *13*, 198–206.
124. Li, W.; Liu, S.; Chen, Y.; Weng, R.; Zhang, K.; He, X.; He, C. Circulating Exosomal microRNAs as Biomarkers of Systemic Lupus Erythematosus. *Clinics* **2020**, *75*, e1528. https://doi.org/10.6061/clinics/2020/e1528.
125. Solé, C.; Moliné, T.; Vidal, M.; Ordi-Ros, J.; Cortés-Hernández, J. An Exosomal Urinary miRNA Signature for Early Diagnosis of Renal Fibrosis in Lupus Nephritis. *Cells* **2019**, *8*, 773. https://doi.org/10.3390/cells8080773.
126. Perez-Hernandez, J.; Martinez-Arroyo, O.; Ortega, A.; Galera, M.; Solis-Salguero, M.A.; Chaves, F.J.; Redon, J.; Forner, M.J.; Cortes, R. Urinary Exosomal miR-146a as a Marker of Albuminuria, Activity Changes and Disease Flares in Lupus Nephritis. *J. Nephrol.* **2021**, *34*, 1157–1167. https://doi.org/10.1007/s40620-020-00832-y.
127. Zununi Vahed, S.; Nakhjavani, M.; Etemadi, J.; Jamshidi, H.; Jadidian, N.; Pourlak, T.; Abediazar, S. Altered Levels of Immune-Regulatory microRNAs in Plasma Samples of Patients with Lupus Nephritis. *BioImpacts* **2018**, *8*, 177–183. https://doi.org/10.15171/bi.2018.20.
128. Higazi, A.M.; Kamel, H.M.; Nasr, M.H.; Keryakos, H.K.; AbdEl-Hamid, N.M.; Soliman, S.A. Potential Role of Circulating miRNA-146a and Serum Kallikrein 1 as Biomarkers of Renal Disease in Biopsy-Proven Lupus Nephritis Patients. *Egypt. Rheumatol.* **2023**, *45*, 73–80. https://doi.org/10.1016/j.ejr.2022.11.001.
